# High-throughput calculation screening for new silicon allotropes with monoclinic symmetry

**DOI:** 10.1107/S2052252523004207

**Published:** 2023-06-17

**Authors:** Qingyang Fan, Jie Wu, Yingbo Zhao, Yanxing Song, Sining Yun

**Affiliations:** a College of Information and Control Engineering, Xi’an University of Architecture and Technology, Xi’an, Shaanxi Province 710055, People’s Republic of China; b College of Science, Xi’an University of Architecture and Technology, Xi’an, Shaanxi Province 710055, People’s Republic of China; c School of Mechanical and Electrical Engineering, Xi’an University of Architecture and Technology, Xi’an, Shaanxi Province 710055, People’s Republic of China; d School of Microelectronics, Xidian University, Xi’an, Shaanxi Province 710071, People’s Republic of China; e School of Materials Science and Engineering, Xi’an University of Architecture and Technology, Xi’an, Shaanxi Province 710055, People’s Republic of China; Alfred University, USA

**Keywords:** high-throughput calculations, silicon allotropes, monoclinic symmetry, electronic properties, photovoltaic applications, crystal structure prediction, properties of solids, crystal design, density functional theory

## Abstract

A total of 87 new monoclinic silicon allotropes are systematically scanned by a random strategy combined with group and graph theory and high-throughput calculations. The new allotropes include 13 with a direct or quasi-direct band gap and 12 with metallic characteristics, and the rest are indirect band gap semiconductors. More than 30 of these novel monoclinic Si allotropes show bulk moduli greater than or equal to 80 GPa, and three of them show even greater bulk moduli than diamond Si. Only two of the new Si allotropes show a greater shear modulus than diamond Si. The crystal structures, stability (elastic constants, phonon spectra), mechanical properties, electronic properties, effective carrier masses and optical properties of all 87 Si monoclinic allotropes are studied in detail. The electron effective masses *m_l_* of five of the new allotropes are smaller than that of diamond Si. All of these novel monoclinic Si allotropes show strong absorption in the visible spectral region. Taken together with their electronic band gap structures, this makes them promising materials for photovoltaic applications. These investigations greatly enrich the current knowledge of the structure and electronic properties of silicon allotropes.

## Abstract 2

High-throughput calculation screening is presented for 87 new low-energy silicon allotropes with monoclinic symmetry.

## 1. Introduction

Monocrystalline silicon is still the cornerstone of the modern semiconductor and microelectronics industry, but some physical properties of diamond Si limit its application, such as its indirect band gap (Kim *et al.*, 2015 ▸). This limitation renders silicon undesirable for the next generation of efficient platforms for applications such as high-performance transistors (Theis & Solomon, 2010 ▸), light-emitting diodes (Ng *et al.*, 2001 ▸; Fujita, 2013 ▸) and thin-film photovoltaic devices (Botti *et al.*, 2012 ▸). Therefore, searching for silicon allotrope structures with a direct or quasi-direct band gap remains a focus for researchers.

Computer-aided materials science has been widely used in the field of electronic materials simulation (Wang *et al.*, 2021 ▸; Wei *et al.*, 2023 ▸; Zhang *et al.*, 2022 ▸) and there have been many theoretical reports on the design of new silicon materials (Lee *et al.*, 2014 ▸; Su *et al.*, 2022 ▸; Song *et al.*, 2022 ▸; He *et al.*, 2018 ▸; Fan *et al.*, 2023 ▸; Cui *et al.*, 2023 ▸; Wei *et al.*, 2022 ▸; Cheng *et al.*, 2018 ▸) based on density functional theory (Hohenberg & Kohn, 1964 ▸; Kohn & Sham, 1965 ▸). However, most of the new silicon structures still belong to the class of indirect band gap semiconductor materials (Cui *et al.*, 2023 ▸; Wei *et al.*, 2022 ▸; Cheng *et al.*, 2018 ▸). Kim *et al.* (2015 ▸) reported the discovery of a new orthorhombic silicon allotrope, Si_24_, formed through a novel high-pressure precursor process, and found that it possesses a quasi-direct band gap near 1.3 eV. Subsequently, Linghu *et al.* (2017 ▸) attempted to modulate Si_24_ into a direct band gap semiconductor by doping with B, Al, Ga, P and As atoms. However, after doping, the results were not satisfactory due to the quasi-direct band gap semiconductor material produced. In other words, doping with Group III or V elements did not lead to successful modulation of Si_24_ into a direct band gap mater­ial, but the band gap range of doped Si_24_ remains the best for making solar cells. Similar electronic band gap modulation can also be achieved by doping with homologous elements (Zhao *et al.*, 2022 ▸; Fan *et al.*, 2019 ▸; Song *et al.*, 2019 ▸; Fan *et al.*, 2020 ▸; Wang *et al.*, 2017 ▸; Fan *et al.*, 2022 ▸) but there have been few successful cases (Wang *et al.*, 2017 ▸; Fan *et al.*, 2022 ▸).

The traditional methods of discovering new materials represented by the empirical trial-and-error method and density functional theory (DFT) (Hohenberg & Kohn, 1964 ▸; Kohn & Sham, 1965 ▸) cannot keep up with the development of today’s materials science because of their long development cycle, low efficiency and high cost (Curtarolo *et al.*, 2013 ▸). High-throughput computational methods are widely used in materials design, testing and analysis, and in other fields, because of their low computing cost, short development cycle, strong data processing ability and high prediction performance. High-throughput computation can greatly reduce the computing cost and shorten the development cycle. At present, a large number of materials design reports (Olsen *et al.*, 2019 ▸; Zhao *et al.*, 2023 ▸; Shi *et al.*, 2021 ▸; Sun & Schwingen­schlögl, 2021 ▸; Wei *et al.*, 2020 ▸; Al-Fahdi, Rodriguez *et al.*, 2021 ▸; Zhang *et al.*, 2018 ▸; Al-Fahdi, Ouyang & Hu, 2021 ▸; Blatov *et al.*, 2021 ▸; Takagi & Maeda, 2020 ▸) utilize high-throughput computations.

Recently, the configuration space of two-dimensional planar *sp* ^2^ carbon was studied using high-throughput calculations and the RG^2^ method (a random strategy based on a combination of group and graph theory) (Shi *et al.*, 2018 ▸). To be specific, 1114 new carbon allotropes were identified (Shi *et al.*, 2021 ▸), including 241 semiconductors, 683 normal metals and 190 Dirac semimetals, and a set of crystal structure search code using RG^2^ methods was proposed by Shi *et al.* (2018 ▸), which can be used for research into crystal structure solution, crystal structure prediction and design of functional materials of interest in condensed matter physics and computational materials science. Other structure prediction methods or programs such as basin hopping (Wales & Doye, 1997 ▸; Iwamatsu & Okabe, 2004 ▸; White & Mayne, 1998 ▸), *USPEX* (Oganov & Glass, 2006 ▸; Glass *et al.*, 2006 ▸; Oganov *et al.*, 2010 ▸; Lyakhov *et al.*, 2010 ▸; Oganov *et al.*, 2011 ▸; Lyakhov *et al.*, 2013 ▸; Niu *et al.*, 2019 ▸) and *CALYPSO* (Wang *et al.*, 2010 ▸; Wang *et al.*, 2012 ▸) also play a significant role in *ab initio* structure prediction.

In this work, 87 monoclinic silicon allotropes are identified using high-throughput calculations and RG^2^. All 87 of these novel allotropes have lower relative enthalpy values (below 0.300 eV per atom) than diamond Si. According to their electronic band structures, 13 of these new silicon allotropes have a direct or quasi-direct band gap and 12 of them have metallic characteristics.

## 2. Computational methods

**Figure 1:**
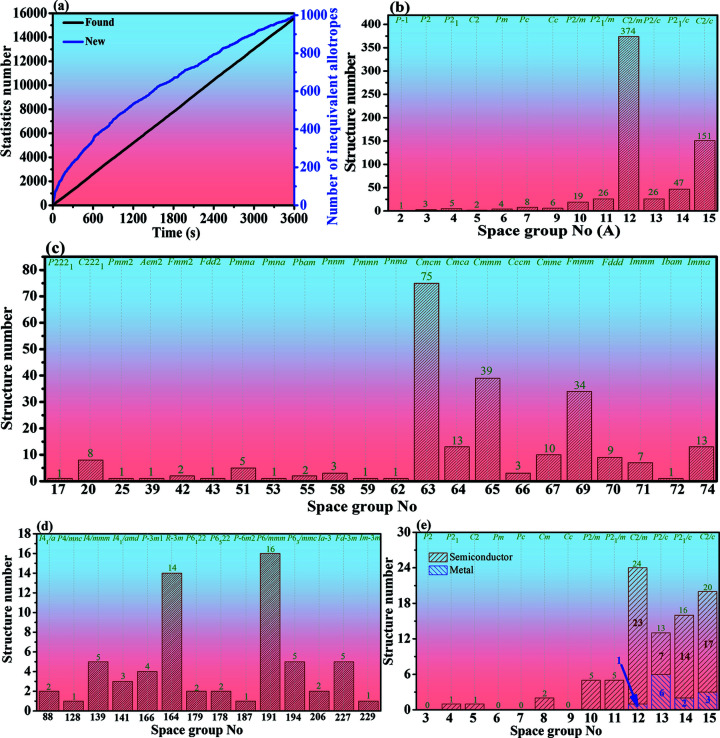
(*a*) The speed and number of new structures generated. (*b*)–(*d*) The number of different space groups in the new structures predicted by RG^2^. (*e*) The distribution of different space groups after optimization.

The space group range we set in RG^2^ was 3–15 for monoclinic symmetry; RG^2^ randomly generated 15 669 initial structures with lattice parameters of 3 Å ≤ *a* ≤ 20 Å, 3 Å ≤ *b* ≤ 30 Å, 3 Å ≤ *c* ≤ 50 Å, 30° ≤ α ≤ 150°, 30° ≤ β ≤ 150° and 30° ≤ γ ≤ 150°. After duplicate structures had been filtered out by RG^2^, a total of 1015 structures of silicon allotropes with monoclinic symmetry remained. Structures that were obviously two-dimensional were then removed, leaving only ∼4.53% (710) of the original 15 699 that can be built into 4-coordinated networks (4-connected quotient graph), which is slightly fewer than obtained by Shi *et al.* (2021 ▸) (∼5%). We only ran the process of predicting the structures for 60 min and the speed of generating new structures is roughly quadratically related to the number of structures. As shown in Fig. 1 ▸(*a*), the generation rate decreases gradually with time. Approximately 710 new three-dimensional silicon structures are in the range of space groups 3–15; that is, approximately 69.95% (of the 1015 monoclinic structures) meet the requirements. Among all the structures predicted by RG^2^, the space group *C*2/*m* (No. 12) is the most common. All the space groups generated by RG^2^ and the number of structures belonging to each space group are shown in Figs. 1 ▸(*b*)–1 ▸(*d*). Then, removing duplicate structures from the set of proposed structures leaves 389 different 3D *sp* ^3^ monoclinic silicon allotropes.

Density functional theory (DFT) (Hohenberg & Kohn, 1964 ▸; Kohn & Sham, 1965 ▸) based on the Cambridge series total energy package (*CASTEP*; Clark *et al.*, 2005 ▸) was used to implement this work. RG^2^ generated thousands of geo­metrically acceptable structures and these were then optimized using *CASTEP*. The Perdew–Burke–Ernzerhof (Perdew *et al.*, 1996 ▸) functional of the generalized gradient approximation (GGA) exchange and correlation functional were used for geometry optimization and property prediction. To describe the interaction between electrons and ions, the ultrasoft quasipotential (Vanderbilt, 1990 ▸) was used in the calculations. The Broyden–Fletcher–Goldfarb–Shanno (Pfrommer *et al.*, 1997 ▸) minimization was used to optimize the geometry. For all monoclinic Si allotropes, the plane wave energy cut-off value was set to 340 eV. The Brillouin zone was sampled with Monkhorst–Pack meshes (Monkhorst & Pack, 1976 ▸) with special *k*-point grids (∼2π × 0.025 Å) for all monoclinic Si allotropes.

The phonon spectra of the monoclinic Si allotropes were studied by density functional perturbation theory (Baroni *et al.*, 2001 ▸). The elastic constants and optical absorption spectra were calculated using *CASTEP* and effective masses were calculated with the *MedeA* Vienna *ab initio* simulation package (*MedeA-VASP*) (Hohenberg & Kohn, 1964 ▸; Kohn & Sham, 1965 ▸; Fonari & Sutton, 2012 ▸; Hafner, 2008 ▸). The bulk modulus, shear modulus and Young’s modulus were estimated by the Voigt–Reuss–Hill approximation method (Voigt, 1928 ▸; Reuss, 1929 ▸; Hill, 1952 ▸). The electronic band structures of all the new monoclinic Si allotropes were calculated utilizing the Heyd–Scuseria–Ernzerhof (HSE06) hybrid functional (Krukau *et al.*, 2006 ▸; Heyd *et al.*, 2003 ▸).

The naming rules for the new structures, taking 8-6-24-232931 as an example, are as follows: the first component 8 of the crystal structure name is the space group number, the second component 6 is the number of non-equivalent positions of silicon atoms in the conventional cell, the third component 24 is the number of atoms in the conventional cell and the fourth component 232931 is an anti-duplication number. Here, a random number representation is used to generate the corresponding initial structure, which ensures that different structures will have different numbers under the same input parameters, and also ensures that the probability of repeating a number for different structures under different input parameters is very low.

## 3. Results and discussion

### 3.1. Structural and topology properties

**Figure 2:**
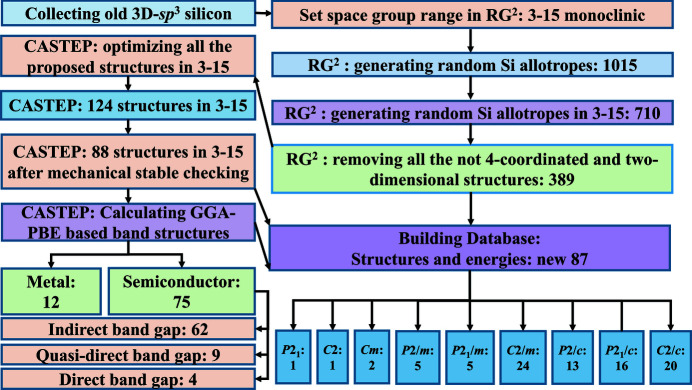
The work flow for high-throughput screening of the crystalline structures and electronic properties of Si allotropes in 3-15 associated with RG^2^.

Because of the variety and importance of 3D silicon allotropes, we have revisited the structural configuration of 3D *sp* ^3^ silicon allotropes in a more systematic way using a high-throughput methodology associated with RG^2^ (Shi *et al.*, 2018 ▸). As shown in Fig. 2 ▸, we first collected the previously reported silicon allotropes and then learnt their fundamental structural features using RG^2^ to build the initial database. Finally, appropriate structures were generated by limiting the coordination numbers of the atoms, the bond length, the bond angle, the space group and other structural information.

First, as shown in Fig. 2 ▸, we used *CASTEP* to optimize the 389 different 3D monoclinic *sp* ^3^ silicon allotrope structures. Through structural optimization by *CASTEP*, some structures become other phases. For instance, some structures are transformed into a variety of hexagonal phases, as proposed recently by Wei *et al.* (2022 ▸), and diamond polytypes, such as 15-2-16-231721, 12-4-16-030405 and 15-2-16-001019, are transformed into 4H or 2H Si structures, 5-6-24-163555 is transformed into a 6H structure, 12-7-28-083128 is transformed into a 7H structure, 15-3-24-070924 is transformed into a 9R structure and 3-4-32-31817 is transformed into a diamond polytype. As a result, 124 structures were able to maintain a monoclinic phase. We then used *CASTEP* to verify the elastic constants. It was found that there are many silicon allotropes which fail to meet the condition of mechanical stability. The final destination of these 389 3D monoclinic *sp* ^3^ silicon allotropes after *CASTEP* optimization is explained in the flow chart in Fig. 2, and all the results are shown in Fig. S1 in the supporting information. Since there are many structures in space groups 12–15, the screening process is complex, and the specific conversion process is shown in Figs. S1(*j*), S1(*k*) and S1(*l*). Taking space group *C*2/*c* (No. 15) as an example, there are 126 structures in space group *C*2/*c* in total. After structural optimization using *CASTEP* based on DFT, 12 structures are repeated, nine structures are scattered (unstable) and three are transferred to space group No. 193. A detailed structural transformation is shown in Fig. S1(*l*). Finally, 20 structures in space group *C*2/*c* were obtained.

After the above geometric structure optimization, elastic constant and phonon spectra calculations were done, also using *CASTEP*. There are 87 different new 3D silicon allotrope structures with monoclinic symmetry (excluding those with structural deformation or space group transformation) whose structures are different from those of monoclinic silicon allotrope structures predicted previously (Wang *et al.*, 2014 ▸; Fan *et al.*, 2016 ▸; Wei *et al.*, 2019 ▸; Lee *et al.*, 2016 ▸; Amsler *et al.*, 2015 ▸). We further studied their structures and physical properties using first-principles calculations. Among them, in terms of electronic band structures, all of the space groups *P*2_1_, *C*2, *Cm*, *P*2/*m* and *P*2_1_/*m* are semiconductor structures, while the space groups *C*2/*m*, *P*2/*c*, *P*2_1_/*c* and *C*2/*c* have both semiconductor and metal structures. The silicon allotropes in *P*2/*c* show the most metallic structures, up to six, while space group *C*2/*m* shows the fewest with metallic properties, having only one structure. Through electronic band structure calculations, we found that among the 87 different new 3D monoclinic *sp* ^3^ silicon allotrope structures, there are 12 with metal properties and 75 with semiconductor properties. Thus, among the 87 new monoclinic 3D silicon structures, approximately 13.79% show metallicity, while approximately 86.36% show semiconductor properties. Among all the structures that show semiconductor properties, 13 were found with a direct or quasi-direct band gap, accounting for about 14.94% of the total structures. In addition, 12-3-12-232409 Si is the same as *m*C12-Si in the work of Wang *et al.* (2014 ▸).

**Figure 3:**
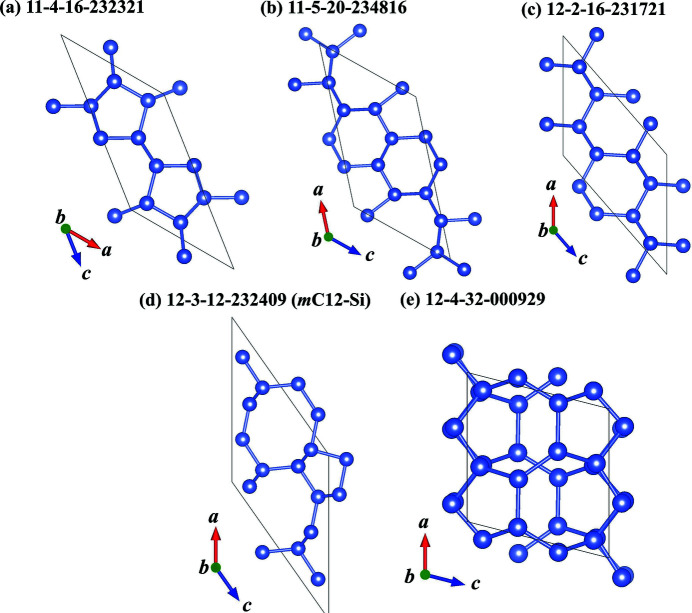
The crystal structures of the five novel monoclinic silicon allotropes with direct band gaps.

The crystal structures of these 87 new 3D *sp* ^3^ silicon allotrope structures are shown in Fig. 3 ▸ and Fig. S2. We selected the 13 new silicon allotropes with a direct or quasi-direct band gap to study their physical properties, and the crystal structures of 12-3-12-232409 Si and of four of the new silicon allotropes with a direct band gap are shown in Fig. 3 ▸. The conventional cells of 11-4-16-232321, 11-5-20-234816, 12-2-16-231721, 12-3-12-232409 (*m*C12-Si) and 12-4-32-000929 are composed of 16, 20, 16, 12 and 32 atoms, respectively. The unit-cell parameters of 11-4-16-232321, 11-5-20-234816, 12-2-16-231721, 12-3-12-232409 (*m*C12-Si) and 12-4-32-000929, together with other novel silicon allotropes with a quasi-direct band gap, are shown in Table 1 ▸. Allotrope 12-3-12-232409 Si exhibits a direct band gap, with equilibrium unit-cell parameters of *a* = 10.755 Å, *b* = 3.855 Å, *c* = 10.981 Å and β = 144.8° in this work, and these are consistent with the *m*C12-Si data which were reported previously (Wang *et al.*, 2014 ▸). The unit-cell parameters for the other novel silicon allotropes with monoclinic symmetry are listed in Table S1.

The topological characteristics of all the monoclinic silicon allotrope structures were calculated using *ToposPro* (Blatov *et al.*, 2014 ▸) on the *Topcryst* website (https://double.topcryst.com/index.php) (Shevchenko *et al.*, 2022 ▸). Among the 87 structures predicted in this work, the topological types of 13 of them are previously unknown. In addition, only a few topological types of silicon structures are similar to those recorded in SACADA (Samara Carbon Allotrope Database; Hoffmann *et al.*, 2016 ▸), such as 4^2^T112 which is the same as the 147th in SACADA, and 4^2^T265 which is the same as the 442nd in SACADA. Although these same topology types are recorded in SACADA, there are still more than 50 silicon allotropes not recorded in SACADA, with orders 22–74 as shown in Table S1.

### 3.2. Stability

It is important to analyse the stability of new materials to understand them better. Therefore, the elastic constants of 13 new silicon allotropes with direct or quasi-direct band gap are listed in Table 2 ▸, and the elastic constants of the other silicon allotropes are shown in Table S2. For stable monoclinic phases, their independent elastic constants *C*_11_, *C*_22_, *C*_33_, *C*_44_, *C*_55_, *C*_66_, *C*_12_, *C*_13_, *C*_15_, *C*_23_, *C*_25_, *C*_35_ and *C*_46_ should obey the following generalized Born mechanical stability criteria (Wu *et al.*, 2007 ▸):

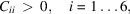




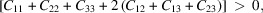




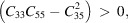




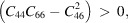




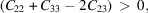




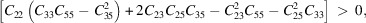




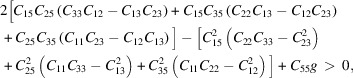




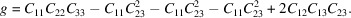




**Figure 4:**
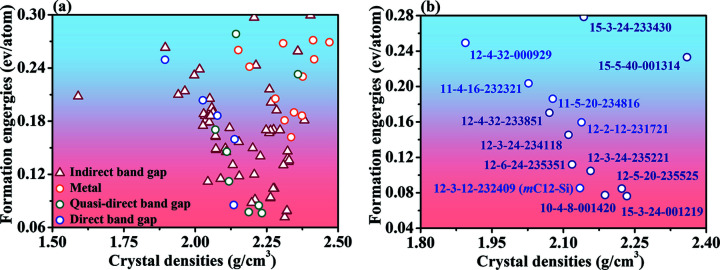
(*a*) The crystal densities and formation energies of all 87 new 3D *sp* ^3^ silicon allotrope structures. (*b*) The detailed crystal densities and formation energies of the 13 monoclinic silicon structures with direct or quasi-direct band gaps.

Table 2 ▸ and Table S2 show that all the calculated elastic constants meet the mechanical stability criteria, and thus all the silicon allotropes mentioned in this work are mechanically stable. The crystal densities and formation energies for all 87 new 3D *sp* ^3^ silicon allotrope structures are shown in Fig. 4 ▸(*a*). The formation energies of all allotropes were calculated by setting the energy of diamond Si to 0. The detailed crystal densities and formation energies for the 13 new silicon allotropes with direct or quasi-direct band gaps are shown in Fig. 4 ▸(*b*). These monoclinic silicon structures with direct or quasi-direct band gaps are mainly clustered in four space groups, *P*2/*m*, *P*2_1_/*m*, *C*2/*m* and *C*2/*c*.

The relative energies of these novel monoclinic silicon structures with direct or quasi-direct band gaps are in the range 0.06–0.18 eV per atom; the relative energies of most of the structures are less than 0.30 eV per atom. Fig. 4 ▸(*a*) shows the crystal densities of all 87 new 3D *sp* ^3^ silicon allotropes, which range from 1.50 to 2.50 g cm^−3^. In addition, from Figs. 4 ▸(*a*) and 4 ▸(*b*), the formation energies of these silicon structures are 85–249 meV per atom, higher than that of diamond Si.

The dynamic stability of these novel monoclinic silicon allotropes was also investigated. The phonon spectra are shown in Fig. S3. No negative frequencies are found throughout the first Brillouin region, indicating that these new monoclinic silicon allotropes are dynamically stable.

### 3.3. Mechanical properties

Table 2 ▸ also shows the bulk modulus, shear modulus and Young’s modulus of 13 new silicon allotropes with direct or quasi-direct band gaps and of diamond Si. The bulk moduli for 13 new silicon allotropes with direct or quasi-direct band gaps are in the range 63–80 GPa, and the shear moduli are in the range 27–55 GPa. The other elastic constants and the bulk modulus, shear modulus and Young’s modulus are shown in Table S2. From Table 2 ▸ and Table S2, the bulk moduli of these monoclinic silicon allotropes range from 42 to 99 GPa, and the shear moduli range from 27 to 66 GPa. The mechanical properties of some of these new structures are better than those of diamond silicon. The silicon allotrope 13-3-12-232508 has the greatest bulk modulus and 15-3-24-232448 has the greatest shear modulus. The bulk moduli of three new structures (13-3-12-232508, 14-5-20-232541 and 15-3-24-232448) are greater than that of diamond Si. The shear moduli of two new structures (14-4-16-232446 and 15-3-24-232448) are greater than or equal to 60 GPa, and thus very close to the value for diamond Si.

**Figure 5:**
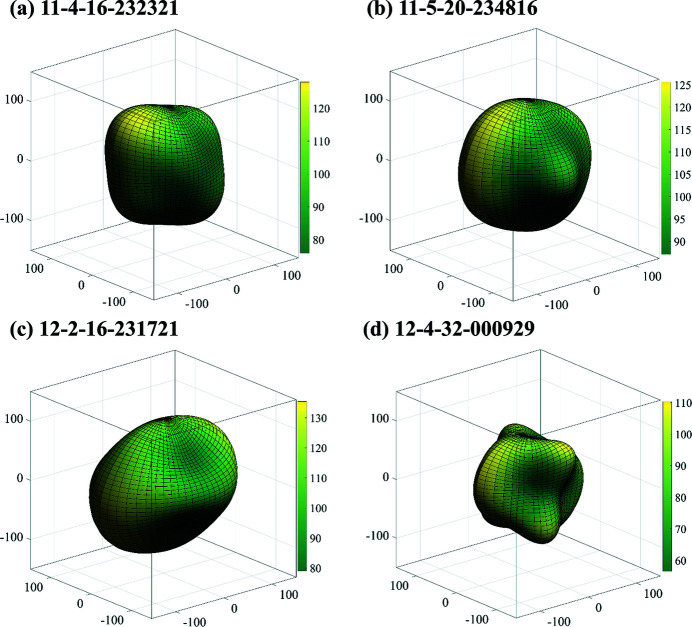
Three-dimensional plots of the Young’s moduli for the four new monoclinic silicon allotropes with direct band gaps.

The 3D directional dependence of the Young’s modulus for an isotropic structure can be illustrated as a sphere, while deviation from a sphere represents anisotropy (Xing & Li, 2023*a* ▸,*b* ▸; Yu *et al.*, 2022 ▸; Liu *et al.*, 2023 ▸). Therefore, to observe the anisotropy of these novel monoclinic silicon allotropes with direct or quasi-direct band gaps more directly, the 3D directional dependence of the Young’s moduli for the 13 silicon allotropes with direct and quasi-direct band gaps were studied, and the results are shown in Fig. 5 ▸ and Fig. S4. All the 3D directional dependences of the Young’s moduli deviate from spheres, so they all show different degrees of anisotropy. The maximum values *E*_max_ (the minimum values *E*_min_) for 11-4-16-232321, 11-5-20-234816, 12-2-16-231721, 12-3-12-232409 (*m*C12-Si) and 12-4-32-000929 are 127.98 GPa (75.93 GPa), 125.61 GPa (87.08 GPa), 135.45 GPa (79.03 GPa), 178.83 GPa (87.84 GPa) and 110.39 GPa (56.61 GPa), respectively. The difference between the *E*_max_/*E*_min_ ratio and 1 reflects the degree of anisotropy of the crystal. The *E*_max_/*E*_min_ ratios of the Young’s moduli for 11-4-16-232321, 11-5-20-234816, 12-2-16-231721, 12-3-12-232409 (*m*C12-Si) and 12-4-32-000929 are 1.69, 1.44, 1.71, 2.04 and 1.95, respectively. The results for monoclinic silicon allotropes with a quasi-direct band gap show that the mechanical anisotropy in the Young’s modulus is greatest for 15-3-24-233430 and smallest for 15-3-24-001219 (Fig. S4).

### 3.4. Electronic properties

**Figure 6:**
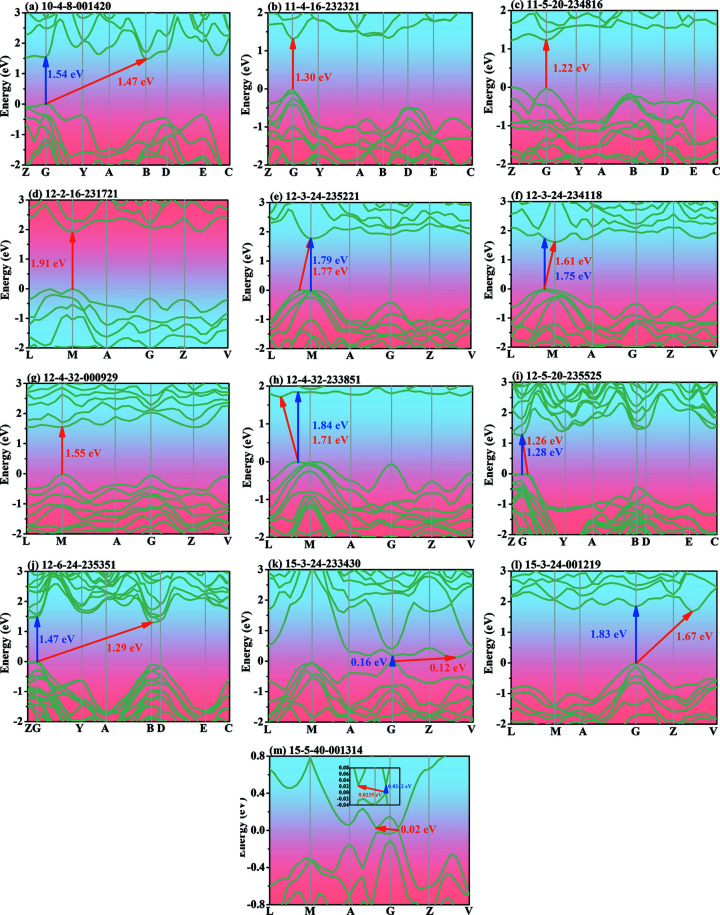
The electron band structures of the 13 novel monoclinic silicon allotropes with direct or quasi-direct band gaps.

The electronic band structure types of these new silicon allotropes are summarized in Fig. 1 ▸(*e*), of which 12 are metallic and the remaining 75 are semiconductors. The electronic band structures of 13 novel silicon allotropes with direct or quasi-direct band gaps in monoclinic symmetry are illustrated in Fig. 6 ▸. Because the valence band maximum (VBM) and conduction band minimum (CBM) for four of these new silicon allotropes (11-4-16-232321, 11-5-20-234516, 12-2-16-231721 and 12-4-32-000929) are located on the same positions, these four are classed as direct band gap semiconductor materials, and the other nine are quasi-direct band gap materials. The band gaps for 11-4-16-232321, 11-5-20-234516, 12-2-16-231721 and 12-4-32-000929 are 1.30, 1.22, 1.91 and 1.55 eV, respectively. The electronic band structures of the remaining silicon allotropes with semiconductor or metallic properties are shown in Figs. S5 and S6, respectively. The difference between the direct and indirect band gaps of silicon allotrope 15-5-40-001314 is very small (detailed in Fig. 6 ▸) and the difference for silicon allotrope 12-6-24-235351 is the largest (0.18 eV), but still slightly smaller than that of Q736 (0.19 eV) (Lee *et al.*, 2014 ▸, 2016 ▸). Although these silicon allotropes have different band gaps and band gap types, they are suitable for optoelectronic devices, especially 11-4-16-232321, 11-5-20-234816, 12-5-20-235525 and 12-6-24-235351: due to their band gap ranges (1.0–1.5 eV), these are ideally suited for solar cells (Lewis, 2007 ▸). For the 12 metallic silicon allotropes, either the conduction band or the valence band crosses the Fermi level.

The effective mass of the carrier has a great influence on the transport performance of a semiconductor material. To understand and study the transport properties of these silicon allotropes with direct band gap more directly and effectively, their effective masses were studied. The effective mass (*m*) can be calculated as follows (Bardeen & Shockley, 1950 ▸):

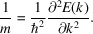

**Figure 7:**
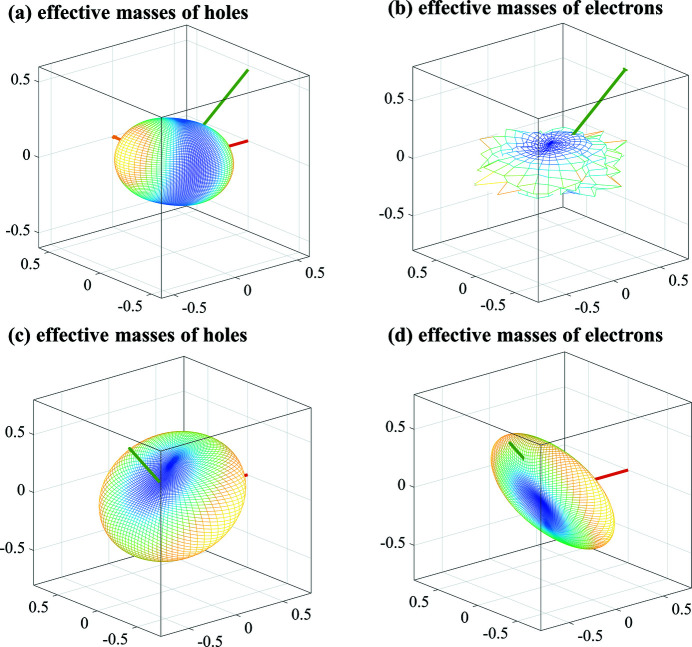
The 3D contours of the effective masses (*m*_0_) of holes and electrons, respectively, for (*a*)–(*b*) 11-4-16-232321 and (*c*)–(*d*) 11-5-20-234816.

The 3D directional dependence of the electron and hole effective masses of 11-4-16-232321 and 11-5-20-234816 are shown in Fig. 7 ▸. Table 3 ▸ lists the hole effective masses of 11-4-16-232321, 11-5-20-234816 and diamond Si. As seen in Fig. 7 ▸, all the 3D shapes of the hole effective masses of 11-4-16-232321 and 11-5-20-234816 exhibit anisotropy. As given in Table 3 ▸, the theoretical values for the hole effective masses for diamond Si are in excellent agreement with the experimental results for heavy holes. The hole effective masses of 11-4-16-232321 and 11-5-20-234816 are slightly larger than that of diamond Si.

The electron effective masses of 11-4-16-232321, 11-5-20-234816, 12-2-16-231721, 12-3-12-232409 (*m*C12-Si) and diamond Si are listed in Table 4 ▸. The electron effective masses *m_l_* of 11-4-16-232321, 11-5-20-234816, 12-2-16-231721 and 12-3-12-232409 (*m*C12-Si) are smaller than that of diamond Si, with the value for 11-5-20-234816 being approximately two-thirds that of diamond Si, the values for 11-4-16-232321 and 12-2-16-231721 being less than one-third of that of diamond Si and the value for 12-3-12-232409 (*m*C12-Si) being approximately one-third that of diamond Si. In addition, the electron effective masses *m_t_*_2_ of 11-4-16-232321 and 11-5-20-234816 are slightly smaller than that of diamond Si.

**Figure 8:**
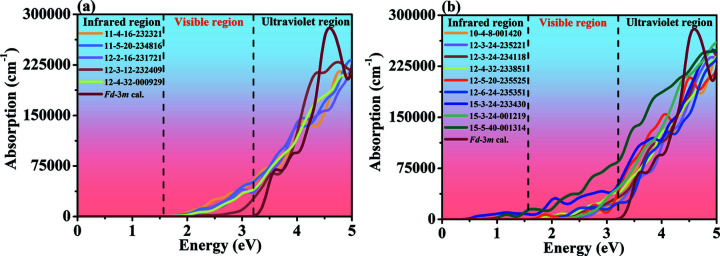
The absorption spectra for monoclinic silicon allotropes with (*a*) direct and (*b*) quasi-direct band gaps. The absorption spectra were calculated using the HSE06 hybrid functional.

The absorption spectra for the new monoclinic silicon allotropes with direct and quasi-direct band gaps are shown in Fig. 8 ▸, and the absorption spectra for the new monoclinic silicon allotropes with indirect band gaps are shown in Fig. S7. As can be seen in Fig. 8 ▸ and Fig. S7, the absorption spectra for these monoclinic silicon allotropes with direct and quasi-direct band gaps are stronger than that of diamond Si in the visible region. From Fig. 8 ▸(*b*), it can be observed that the monoclinic silicon allotropes with quasi-direct band gaps show better photon absorption performance than diamond Si, which is due to the fact that all their absorption spectra in the visible range have a higher magnitude than that of diamond Si. These results also suggest that our proposed silicon allotropes with quasi-direct band gaps start to absorb sunlight at lower energies than diamond Si, as do those with direct band gaps proposed in this work. The novel silicon allotropes with indirect band gaps also have better light absorption capacity than diamond Si. From what has been discussed above for Fig. 8 ▸ and Fig. S7, it is concluded that these monoclinic silicon allotropes show promising prospects for photovoltaic applications.

## 4. Conclusions

In total, 87 new monoclinic silicon allotropes have been obtained and confirmed by RG^2^ and high-throughput calculations. The vast majority (more than 86%) of these allotropes are in space groups *C*2/*m*, *P*2/*c*, *P*2_1_/*c* and *C*2/*c*. Crystal structures in space group *P*2/*c* are the greatest in number, while crystal structures in space groups *P*2, *Pm* and *Cc* were not found in this work. This is probably due to the fact that we only used RG^2^ for 60 min, which is not enough time to generate more structures in space groups *P*2, *Pm*, *Pc* and *Cc*.

All the elastic constants for the new monoclinic silicon allotropes proposed in this work meet the Born mechanical stability criteria. Among the 87 allotropes presented here, 13-3-12-232508 silicon shows the greatest bulk modulus (99 GPa) and 15-3-24-232448 silicon shows the greatest shear modulus (66 GPa).

Four silicon allotropes, 11-4-16-232321, 11-5-20-234816, 12-2-16-231721 and 12-4-32-000929, exhibit direct band gap characteristics and nine of the allotropes exhibit quasi-direct band gap characteristics. For the allotropes with direct band gaps, the electron effective masses *m_l_* of 11-4-16-232321, 11-5-20-234816, 12-2-16-231721 and 12-3-12-232429 are smaller than that of diamond Si, while the electron effective masses *m*_*t*2_ of 11-4-16-232321 and 11-5-20-234816 are also smaller than that of diamond Si.

All 74 novel monoclinic silicon allotropes with semiconductor properties have a strong absorption capacity in the visible region, which shows great potential for application in optoelectronic devices.

## Supplementary Material

Additional tables and figures. DOI: 10.1107/S2052252523004207/ct5018sup1.pdf

CIFs for the new allotropes. DOI: 10.1107/S2052252523004207/ct5018sup2.zip

## Figures and Tables

**Table 1:** The crystal lattice parameters (*a*, *b*, *c* in Å; β in °) and formation energies (eV atom^−1^) of monoclinic silicon allotropes with direct and quasi-direct band gaps and diamond Si

Allotrope	Topology	*a*	*b*	*c*	β	ρ	Δ*E*
10-4-8-001420	4^4^T35	6.285	3.886	8.207	58.3	2.187	0.077
11-4-16-232321	4^4^T34597-HZ	7.443	6.801	11.765	38.2	2.027	0.204
11-5-20-234816	4^5^T238323-HZ	11.615	6.679	7.688	131.2	2.078	0.186
12-2-16-231721	4^2^T168	7.753	6.885	10.293	139.2	2.138	0.163
12-3-12-232409	NSI	10.755	3.855	10.981	144.8	2.135	0.085
*m*C12-Si†	NSI	10.75	3.86	10.95	144.7	2.13	
12-3-24-235221	4^3^T142	7.739	6.660	11.639	120.1	2.157	0.105
12-3-24-234118	4^3^T162-CA	7.751	6.786	13.957	133.8	2.111	0.146
12-4-32-000929	4^4^T36059-HZ	7.443	14.948	7.309	104.3	1.894	0.249
12-4-32-233851	4^4^T36746-HZ	7.766	6.878	14.045	106.1	2.071	0.170
12-5-20-235525	4^5^T236809-HZ	17.882	3.888	19.720	162.2	2.222	0.085
12-6-24-235351	4^6^T1884-HZ	18.922	3.882	21.651	160.6	2.118	0.112
15-3-24-233430	3,4T90	7.327	6.555	15.372	135.0	2.233	0.279
15-3-24-001219	4^3^T3652-HZ	11.109	6.403	11.303	141.4	2.139	0.076
15-5-40-001314	Unknown	8.996	7.578	12.978	116.7	2.360	0.233
Diamond Si‡	Diamond	5.442					0
Diamond Si§		5.341					

† Wang *et al.* (2014 ▸).

‡ Fan *et al.* (2016 ▸).

§ Lide (1994 ▸), experimental data.

**Table 2:** Elastic constants (GPa) and elastic moduli (GPa) of monoclinic silicon allotropes with direct and quasi-direct band gaps, and diamond Si

Allotrope	*C*_11_	*C*_12_	*C*_13_	*C*_15_	*C*_22_	*C*_23_	*C*_25_	*C*_33_	*C*_35_	*C*_44_	*C*_46_	*C*_55_	*C*_66_	*B*	*G*	*E*
10-4-8-001420	153	35	41	−1	164	45	13	156	−3	55	15	55	52	79	55	134
11-4-16-232321	135	43	53	−3	97	42	3	128	−6	33	−5	48	32	69	37	94
11-5-20-234816	149	39	48	−1	118	35	2	131	−4	34	−2	47	33	71	40	101
12-2-16-231721	158	47	47	4	114	38	−2	130	0	29	−1	49	43	73	41	104
12-3-12-232409 (*m*C12-Si)	141	44	54	−6	157	29	0	152	−25	36	−8	57	60	77	50	123
12-3-24-235221	150	48	47	2	136	44	1	152	8	44	6	53	36	79	46	116
12-3-24-234118	136	50	45	−1	93	43	3	160	4	43	2	44	27	72	38	97
12-4-32-000929	126	33	41	−2	92	49	11	113	−2	41	5	39	23	63	33	84
12-4-32-233851	135	45	49	0	98	43	−4	137	7	36	−1	43	32	71	37	95
12-5-20-235525	143	50	37	5	160	32	10	166	3	44	11	54	66	78	55	134
12-6-24-235351	139	34	50	−5	146	45	−4	136	3	59	−3	51	35	75	48	119
15-3-24-233430	144	40	49	24	132	36	9	110	14	22	22	52	33	68	27	72
15-3-24-001219	159	33	40	12	172	39	5	174	2	48	−1	52	43	80	54	132
15-5-40-001314	163	42	40	1	127	48	4	126	8	49	3	54	56	74	50	122
Diamond Si†	154	56								79				88	64	155
Diamond Si‡	166	64								80						

† Fan *et al.* (2016 ▸).

‡ Pfrommer *et al.* (1997 ▸), experimental data.

**Table 3:** Effective masses (*m*_0_) of holes for two of the new silicon allotropes with direct band gaps, and for diamond Si, along different direction vectors

	Hole effective mass		
Allotrope	*m*_*l*_ direction	*m*_*t*1_ direction	*m*_*t*2_ direction
11-4-16-232321	0.424	0.286	0.205
11-5-20-234816	0.729	0.319	0.294
Diamond Si†	0.268 (heavy)	0.268 (heavy)	0.268 (heavy)
Diamond Si‡	0.260 (heavy)	0.260 (heavy)	0.260 (heavy)
Diamond Si§	0.310 (heavy)	0.310 (heavy)	0.310 (heavy)
Diamond Si¶	0.460 (heavy)	0.460 (heavy)	0.460 (heavy)
Diamond Si†	0.178 (light)	0.178 (light)	0.178 (light)
Diamond Si‡	0.260 (light)	0.260 (light)	0.260 (light)
Diamond Si§	0.227 (light)	0.227 (light)	0.227 (light)
Diamond Si¶	0.171 (light)	0.171 (light)	0.171 (light)

† This work.

‡ Ramos *et al.* (2001 ▸).

§ Wang *et al.* (2006 ▸).

¶ Dexter & Lax (1954 ▸), experimental data.

**Table 4:** Effective masses of electrons for silicon allotropes with direct band gaps, and for diamond Si, along different direction vectors (in *m*_0_)

	Electron effective mass		
Allotrope	*m*_*l*_ direction	*m*_*t*1_ direction	*m*_*t*2_ direction
11-4-16-232321	0.297	0.297	0.171
11-5-20-234816	0.680	0.362	0.172
12-2-16-231721	0.289	0.255	0.254
12-3-12-232409	0.317	0.210	0.184
Diamond Si†	0.926	0.195	0.195
Diamond Si†	0.916	0.191	0.191

† This work.

‡ Dexter & Lax (1954 ▸), experimental data.
