# Supplementary material for: High-throughput calculation screening for new silicon allotropes with monoclinic symmetry
Source: IUCrJ. 2023 Jun 17;10(Pt 4):464–74. doi: 10.1107/S2052252523004207 (PMC10324479; doi:10.1107/S2052252523004207)
Supplement: Supplementary file 1 [file m-10-00464-sup1.pdf]

IUCrJ

Volume 10 (2023)

Supporting information for article:

High-throughput calculation screening for new silicon allotropes with monoclinic symmetry

Qingyang Fan, Jie Wu, Yingbo Zhao, Yanxing Song and Sining Yun

Table S1 The crystal lattice parameters (a, b, c in Å; β in °), formation energies (eV/atom), and E_g^i: the band gap of the indirect band gap semiconductor of 74 novel silicon allotropes in monoclinic symmetry.

Order	Name	Topology	Space group	a	b	c	β	ΔE	E_g^i
1	11-4-16-232635	Unknown	$P2_1/m$	10.838	6.505	5.905	56.3	0.118	1.48
2	13-3-12-232717	Unknown	$P2/c$	3.701	10.780	6.021	74.9	0.271	Metal
3	13-4-16-235907	Unknown	$P2/c$	8.608	3.747	12.594	50.6	0.230	Metal
4	13-6-24-233744	Unknown	$P2/c$	9.602	3.764	13.371	80.8	0.190	Metal
5	14-3-12-001732	Unknown	$P2_1/c$	8.599	4.433	7.155	107.5	0.260	Metal
6	14-4-16-232446	Unknown	$P2_1/c$	3.837	6.539	12.821	89.8	0.079	1.43
7	14-4-16-233726	Unknown	$P2_1/c$	5.829	8.616	7.405	117.5	0.093	1.84
8	14-4-16-234845	Unknown	$P2_1/c$	5.823	8.630	10.511	141.4	0.094	1.85
9	14-4-16-235340	Unknown	$P2_1/c$	13.351	6.552	12.804	163.3	0.079	1.47
10	14-5-20-232541	Unknown	$P2_1/c$	10.556	10.492	9.983	159.5	0.300	0.10
11	15-4-32-233356	Unknown	$C2/c$	13.330	7.180	6.802	100.1	0.136	0.89
12	15-4-32-000249	Unknown	$C2/c$	7.072	15.242	6.426	111.0	0.268	Metal
13	2-3-6-071501	4^2T112	$C2/c$	5.193	12.838	3.811	84.6	0.104	1.47
14	15-2-12-011815	4^2T112	$C2/c$	5.191	12.852	3.813	75.4	0.104	1.33
15	5-4-16-232315	4^4T112	$C2$	13.658	3.805	6.513	108.4	0.138	1.13
16	12-3-16-232333	4^3T85	$C2/m$	7.203	7.714	10.937	146.2	0.092	1.17
17	12-4-32-001357	4^4T130	$C2/m$	13.396	6.559	10.236	131.0	0.088	1.91
18	13-2-8-002729	4^2T286	$P2/c$	3.758	7.069	6.670	115.6	0.162	Metal
19	15-2-16-231909	4^2T10	$C2/c$	10.228	5.092	14.019	150.4	0.162	1.81
20	15-2-16-232558	4^2T10	$C2/c$	10.236	5.091	10.784	140.1	0.164	1.80
21	15-2-16-231933	4^2T265	$C2/c$	6.156	14.247	3.843	100.0	0.170	0.78
22	4-6-12-000235	4^6T4102-HZ	$P2_1$	9.787	6.582	3.828	78.8	0.072	1.41
23	8-6-24-232931	4^6T4983-HZ	Cm	7.758	6.768	14.006	133.7	0.149	1.76
24	8-6-24-235459	4^6T4966-HZ	Cm	7.716	7.049	11.694	120.0	0.186	1.48
25	10-3-10-234853	4^3T3824-HZ	$P2/m$	3.803	8.690	6.257	85.1	0.169	0.14
26	10-3-10-001422	4^3T3824-HZ	$P2/m$	6.306	8.666	3.792	84.9	0.167	0.61
27	10-4-16-233217	4^4T38070-HZ	$P2/m$	7.605	3.907	6.565	69.3	0.112	1.23
28	10-4-16-233838	4^4T38070-HZ	$P2/m$	8.106	3.907	6.564	118.6	0.112	1.23
29	11-3-12-232445	4^3T3792-HZ	$P2_1/m$	6.987	6.811	5.840	92.2	0.239	1.20
30	11-3-12-235011	4^3T3792-HZ	$P2_1/m$	9.177	6.777	7.104	140.6	0.232	1.38
31	12-2-16-234627	4^2T447-HZ	$C2/m$	7.467	13.390	6.304	149.9	0.259	0.60
32	12-2-16-235920	4^2T440-HZ	$C2/m$	10.862	6.893	5.404	89.6	0.275	1.37
33	12-2-24-232652	4^2T20-CA	$C2/m$	5.724	10.980	3.703	103.2	0.269	Metal
34	12-3-12-000320	4^3T3538-HZ	$C2/m$	4.345	15.197	3.855	103.9	0.201	0.32

35	12-3-24-235436	4^3T4429-HZ	*C*2/*m*	7.468	13.974	5.604	68.6	0.192	1.22
36	12-3-24-232650	4^3T3703-HZ	*C*2/*m*	7.630	14.085	5.652	110.2	0.214	1.69
37	12-3-24-000329	4^3T149-CA	*C*2/*m*	11.473	6.538	11.176	140.3	0.115	1.97
38	12-3-24-232006	4^3T161-CA	*C*2/*m*	7.691	7.081	11.613	119.7	0.178	1.82
39	12-3-24-234019	4^3T153-CA	*C*2/*m*	13.516	6.960	6.883	60.5	0.263	1.06
40	12-3-24-235933	4^3T160-CA	*C*2/*m*	7.726	7.052	11.642	119.6	0.188	1.46
41	12-3-24-000121	4^3T162-CA	*C*2/*m*	7.734	6.802	11.650	120.0	0.145	1.62
42	12-3-24-001050	4^3T161-CA	*C*2/*m*	7.717	7.062	11.582	119.6	0.182	1.80
43	12-5-40-232334	4^5T241141-HZ	*C*2/*m*	7.803	6.851	17.781	109.0	0.131	1.61
44	12-5-40-235831	4^5T240503-HZ	*C*2/*m*	7.828	7.037	17.983	110.0	0.179	1.46
45	12-5-40-000356	4^5T240498-HZ	*C*2/*m*	7.712	7.531	19.471	119.3	0.210	1.62
46	13-2-8-231921	4^2T1-CA	*P*2/*c*	5.056	5.274	7.230	109.4	0.186	1.12
47	13-3-12-233948	4^3T3400-HZ	*P*2/*c*	5.568	7.024	7.391	109.8	0.195	1.07
48	13-3-12-235231	4^3T4482-HZ	*P*2/*c*	8.574	5.782	7.273	49.2	0.206	1.50
49	13-3-12-235342	4^3T4099-HZ	*P*2/*c*	9.964	5.700	9.035	150.4	0.297	0.75
50	13-3-12-232304	4^3T4123-HZ	*P*2/*c*	6.847	3.767	10.137	110.2	0.205	Metal
51	13-3-12-233214	4^3T4586-HZ	*P*2/*c*	10.066	5.669	7.186	140.7	0.157	1.29
52	13-3-12-232202	4^3T4119-HZ	*P*2/*c*	3.774	10.919	7.254	126.3	0.146	0.34
53	13-3-12-232508	4^3T4117-HZ	*P*2/*c*	9.813	3.760	6.652	73.8	0.186	Metal
54	13-4-16-001718	4^4T37589-HZ	*P*2/*c*	3.792	14.324	6.984	120.6	0.192	0.86
55	14-2-8-232118	4^2T171	*P*2_1/*c*	5.354	5.086	7.249	114.3	0.148	1.08
56	14-2-8-232552	4^2T171	*P*2_1/*c*	7.013	5.089	10.156	150.2	0.149	1.11
57	14-3-12-235423	4^3T3362-HZ	*P*2_1/*c*	5.169	8.950	6.543	122.9	0.150	1.73
58	14-3-12-231751	4^3T59-CA	*P*2_1/*c*	8.385	6.478	7.593	143.4	0.171	0.21
59	14-3-12-235501	4^3T59-CA	*P*2_1/*c*	5.094	6.485	7.601	100.0	0.170	0.23
60	14-4-16-000223	4^4T34646-HZ	*P*2_1/*c*	5.176	5.038	13.495	89.9	0.172	0.74
61	14-4-16-000321	4^4T34671-HZ	*P*2_1/*c*	13.311	5.123	9.326	147.6	0.242	Metal
62	14-4-16-232843	4^4T37972-HZ	*P*2_1/*c*	5.331	9.991	10.515	138.9	0.175	1.35
63	14-4-16-232103	4^4T15-CA	*P*2_1/*c*	6.540	3.802	13.067	80.8	0.134	0.88
64	14-4-16-235403	4^4T38129-HZ	*P*2_1/*c*	8.685	5.789	7.264	67.8	0.120	1.94
65	15-2-16-232050	4^2T1092-HZ	*C*2/*c*	3.724	14.828	7.286	129.9	0.250	Metal
66	15-3-16-234156	4^3T3654-HZ	*C*2/*c*	3.877	18.500	5.402	123.6	0.181	Metal
67	15-3-20-001715	4^3T3324-HZ	*C*2/*c*	10.362	8.476	7.224	138.7	0.141	1.96
68	15-3-20-232008	4^3T3648-HZ	*C*2/*c*	5.294	12.843	9.100	137.1	0.243	0.09
69	15-3-24-232148	4^3T3378-HZ	*C*2/*c*	13.958	3.786	13.727	137.8	0.170	0.75
70	15-3-24-233315	4^3T3660-HZ	*C*2/*c*	10.340	11.489	6.795	142.2	0.216	0.04
71	15-3-24-232448	4^3T4595-HZ	*C*2/*c*	19.122	3.754	6.650	100.2	0.181	0.39
72	15-4-32-235759	4^4T35233-HZ	*C*2/*c*	10.403	10.247	7.289	68.6	0.190	1.98

73	15-4-32-233050	4^4T35333-HZ	*C*2/*c*	7.311	7.332	14.042	105.2	0.186	1.12
74	15-4-32-000324	4^4T39346-HZ	*C*2/*c*	3.796	14.375	12.055	100.7	0.130	0.36

Table S2 The elastic constants (GPa) and elastic moduli (GPa) of 74 novel silicon allotropes.

Order	Name	C_{11}	C_{12}	C_{13}	C_{15}	C_{22}	C_{23}	C_{25}	C_{33}	C_{35}	C_{44}	C_{46}	C_{55}	C_{66}	B	G
1	2-3-6-071501	189	52	37	-2	165	49	6	133	-8	51	3	57	70	83	57
2	4-6-12-000235	160	42	37	-3	160	48	3	181	-2	63	0	53	40	84	55
3	5-4-16-232315	191	34	31	-21	189	43	10	159	-18	61	6	51	51	82	59
4	8-6-24-232931	154	43	51	3	104	43	0	140	2	33	2	49	39	73	41
5	8-6-24-235459	132	46	52	2	72	46	1	134	-3	28	1	46	34	66	33
6	10-3-10-234853	169	39	31	7	161	42	-5	156	-7	39	-1	51	46	79	51
7	10-3-10-001422	116	50	14	-7	155	42	-5	159	4	38	-7	47	25	70	41
8	10-4-16-233217	100	23	31	2	154	60	-5	138	1	60	-2	29	39	67	42
9	10-4-16-233838	96	24	34	8	155	56	-5	149	1	59	-3	39	39	67	45
10	11-3-12-232445	116	35	48	-5	118	48	7	137	-9	32	-10	47	29	69	36
11	11-3-12-235011	136	45	45	3	117	26	6	160	-4	30	8	47	33	71	40
12	11-4-16-232635	139	35	43	9	159	37	1	166	4	41	4	47	39	77	48
13	12-2-16-234627	199	41	44	4	166	37	-5	129	14	25	-1	59	47	81	48
14	12-2-16-235920	111	37	34	4	80	39	11	119	6	35	5	29	21	57	29
15	12-2-24-232652	154	52	49	-4	141	26	-3	205	-9	38	-8	76	63	83	58
16	12-3-12-000320	183	54	38	8	136	54	-6	95	3	50	7	44	63	76	47
17	12-3-16-232333	147	45	43	1	149	40	11	159	-3	50	12	54	55	79	53
18	12-3-24-235436	137	30	39	11	106	56	-8	137	-3	46	-5	45	28	69	39
19	12-3-24-232650	116	28	41	0	100	53	7	138	6	37	5	44	23	65	35
20	12-3-24-000329	134	35	55	-13	152	33	1	139	-13	37	-6	56	36	74	44
21	12-3-24-232006	142	59	50	0	78	34	-2	110	1	26	-1	45	43	65	34
22	12-3-24-234019	120	36	52	-13	63	30	-5	139	-6	29	-7	36	26	57	30
23	12-3-24-235933	87	47	36	6	86	33	15	135	-7	30	-1	64	38	59	36
24	12-3-24-000121	153	45	48	7	112	50	-1	132	5	31	8	54	42	75	41
25	12-3-24-001050	130	42	39	-1	94	52	-5	150	-1	41	-7	46	30	70	38
26	12-4-32-001357	168	34	53	1	139	59	-1	133	2	33	4	56	46	81	45
27	12-5-40-232334	139	46	46	-1	118	44	-3	163	2	43	0	47	31	76	42
28	12-5-40-235831	141	47	50	5	80	41	0	134	-1	27	6	43	36	67	35
29	12-5-40-000356	128	57	39	-1	76	47	-5	124	-1	29	-5	42	39	66	32
30	13-2-8-002729	179	36	51	-6	144	52	9	176	18	68	5	67	42	85	58
31	13-2-8-231921	107	42	41	7	115	38	0	156	5	35	3	48	30	68	39
32	13-3-12-233948	127	52	34	1	118	25	0	143	0	29	-2	42	49	68	41
33	13-3-12-235231	112	60	32	5	130	46	2	135	2	40	2	41	49	72	41
34	13-3-12-235342	89	35	65	4	159	35	8	110	5	41	7	39	40	69	36
35	13-3-12-232304	117	29	63	9	185	40	-4	162	2	48	-15	62	34	79	47
36	13-3-12-233214	128	42	35	6	168	38	3	139	-14	55	4	41	40	73	47

37	13-3-12-232202	175	35	45	-4	148	44	9	166	-2	51	13	69	53	82	58
38	13-3-12-232508	180	27	47	-10	184	71	-5	267	-11	67	-8	55	27	99	57
39	13-3-12-232717	188	25	41	11	155	47	-4	147	-7	50	-5	57	40	80	53
40	13-4-16-001718	163	41	39	-10	141	50	6	156	-16	45	0	62	46	80	52
41	13-4-16-235907	163	39	35	-7	186	27	8	155	7	34	7	59	62	78	55
42	13-6-24-233744	181	24	34	7	178	40	-1	167	4	66	0	48	29	80	54
43	14-2-8-232118	137	52	28	-8	120	42	-4	108	2	58	-2	35	30	67	39
44	14-2-8-232552	122	45	49	-10	122	47	5	131	14	31	-5	48	55	73	41
45	14-3-12-235423	144	52	41	18	143	37	-3	146	9	46	0	49	37	76	46
46	14-3-12-001732	130	4	38	3	86	-15	1	131	3	46	0	44	41	42	46
47	14-3-12-231751	127	57	56	-12	143	38	-8	140	11	45	-4	55	56	79	47
48	14-3-12-235501	124	57	53	12	145	39	5	145	-10	50	2	60	56	79	49
49	14-4-16-000223	125	55	42	-7	116	40	-1	150	-8	51	4	44	35	74	42
50	14-4-16-000321	145	44	56	-5	99	52	-5	145	-21	27	-9	57	9	75	25
51	14-4-16-232843	104	69	54	6	118	34	7	134	-4	34	4	40	44	74	34
52	14-4-16-232103	172	43	36	2	191	40	6	164	-11	59	5	41	60	85	58
53	14-4-16-232446	186	48	42	-1	166	35	-1	168	2	45	0	62	65	85	60
54	14-4-16-233726	154	54	41	-12	119	39	2	166	1	51	1	65	58	78	54
55	14-4-16-234845	167	39	44	2	123	58	2	141	-5	55	0	67	49	79	52
56	14-4-16-235340	190	44	46	-1	167	45	-2	145	3	45	-1	58	62	86	57
57	14-4-16-235403	137	43	39	-3	154	35	-8	150	11	52	-5	47	47	75	50
58	14-5-20-232541	198	43	56	2	145	63	2	154	-12	35	13	34	45	91	42
59	15-2-12-011815	162	58	50	-15	117	38	4	183	-6	48	-8	68	63	82	55
60	15-2-16-231909	122	46	42	-18	114	38	-5	106	-1	35	15	52	40	65	38
61	15-2-16-232558	115	39	30	5	116	52	0	149	-13	33	9	37	50	69	40
62	15-2-16-231933	99	61	51	-1	140	43	-1	158	6	44	5	49	56	78	44
63	15-2-16-232050	168	38	42	-9	139	41	12	176	-12	60	10	61	33	80	52
64	15-3-16-234156	166	52	37	-19	109	60	6	157	-24	66	-4	43	57	79	47
65	15-3-20-001715	133	49	31	-14	136	48	1	169	-16	49	7	35	53	75	46
66	15-3-20-232008	141	34	25	-1	113	38	3	164	2	36	1	37	48	67	45
67	15-3-24-232148	149	44	59	-9	192	35	4	126	8	53	10	46	48	82	50
68	15-3-24-233315	159	57	25	-7	148	32	4	147	4	30	-5	46	63	75	48
69	15-3-24-232448	185	26	16	9	195	42	0	322	9	70	2	48	37	94	66
70	15-4-32-235759	131	34	46	-6	136	53	-3	109	-2	48	-1	33	23	71	36
71	15-4-32-233050	118	49	44	3	110	52	-10	141	3	43	-3	42	30	73	37
72	15-4-32-233356	188	31	39	-2	177	38	-4	146	2	58	-8	43	53	80	57
73	15-4-32-000249	151	37	47	-9	142	50	4	124	7	46	4	25	17	76	33
74	15-4-32-000324	176	29	37	-6	161	55	6	153	-6	61	-1	50	43	82	54

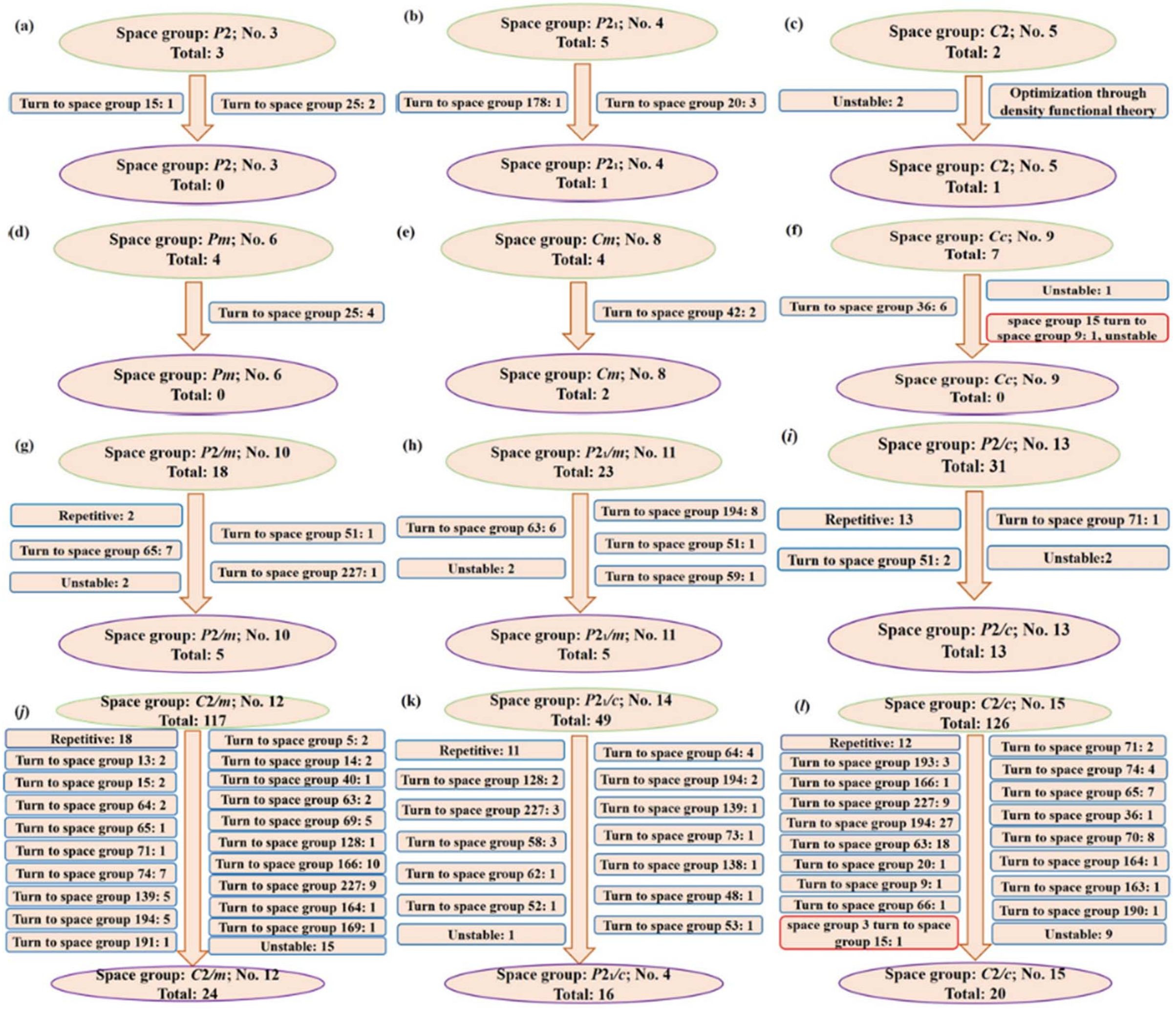

Figure S1 The final destination of these 389 3D sp^3 silicon allotropes in monoclinic symmetry after CASTEP optimization.

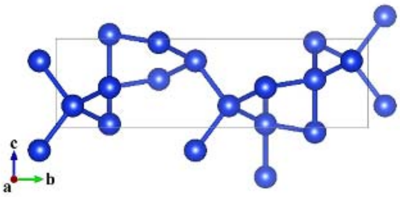

(1) 2-3-6-071501

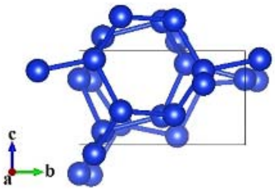

(2) 4-6-12-000235

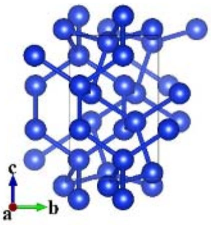

(3) 5-4-16-232315

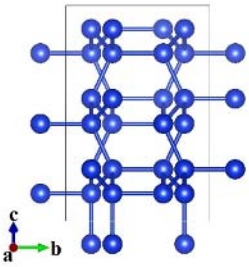

(4) 8-6-24-232931

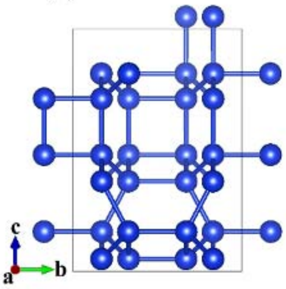

(5) 8-6-24-235459

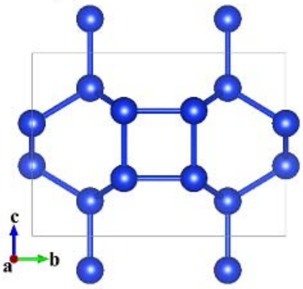

(6) 10-3-10-234853

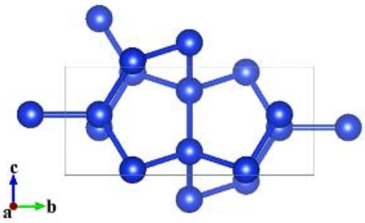

(7) 10-3-10-001422

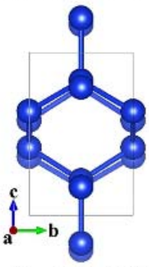

(8) 10-4-16-233217

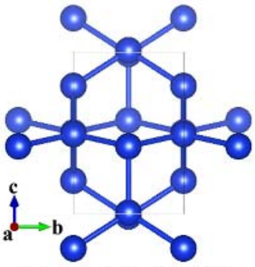

(9) 10-4-16-233838

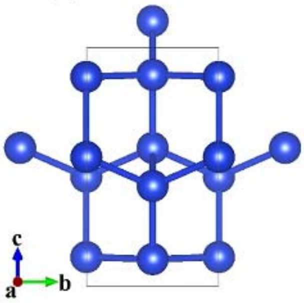

(10) 10-4-8-001420

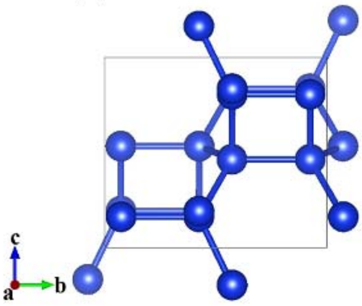

(11) 11-3-12-232445

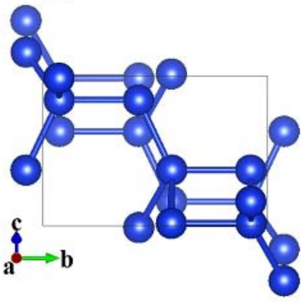

(12) 11-3-12-235011

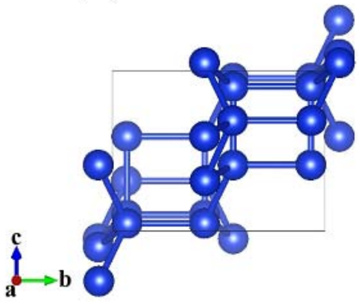

(13) 11-4-16- 232635

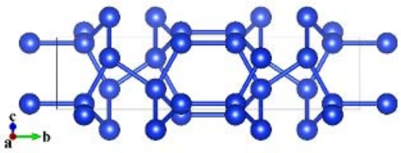

(14) 12-2-16-234627

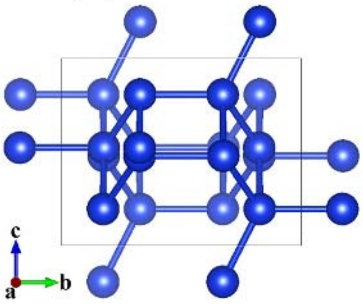

(15) 12-2-16-235920

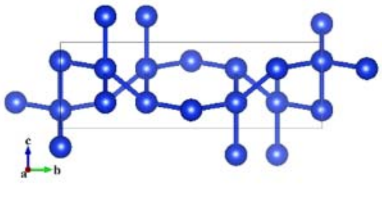

(16) 12-2-24-232652

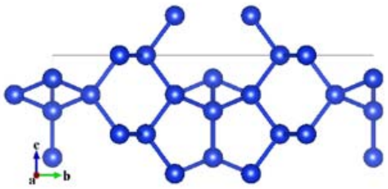

(17) 12-3-12-000320

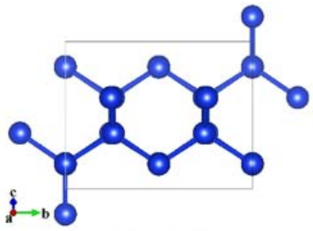

(18) 12-3-16-232333

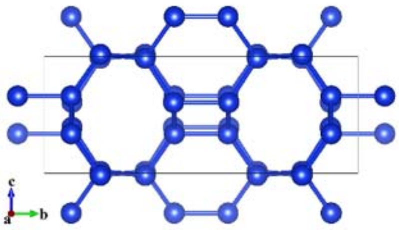

(19) 12-3-24-235436

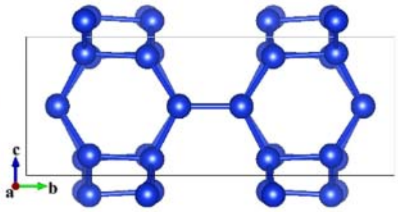

(20) 12-3-24-232650

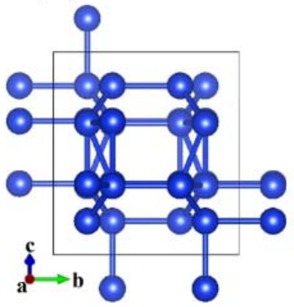

(21) 12-3-24-000329

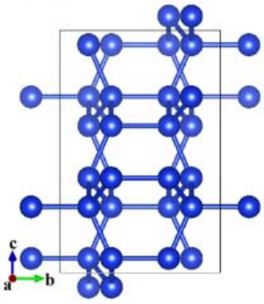

(22) 12-3-24-235221

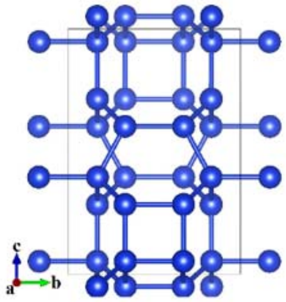

(23) 12-3-24-232006

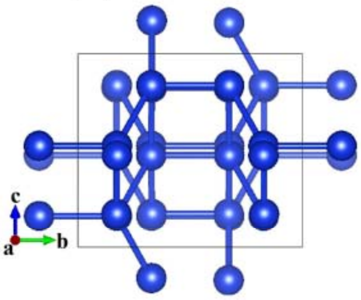

(24) 12-3-24-234019

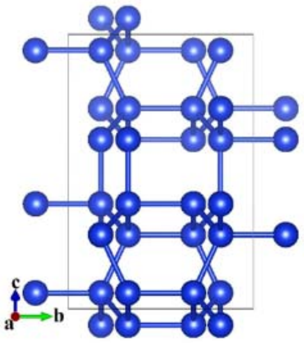

(25) 12-3-24-234118

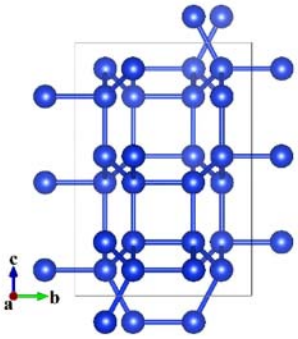

(26) 12-3-24-235933

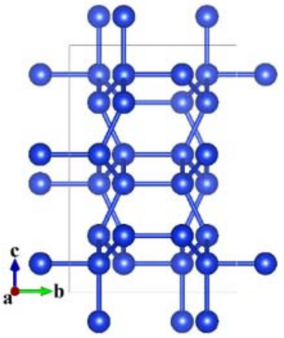

(27) 12-3-24-000121

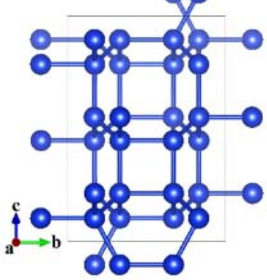

(28) 12-3-24-001050

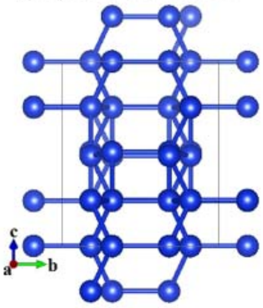

(29) 12-4-32-001357

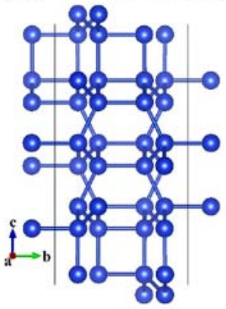

(30) 12-4-32-233851

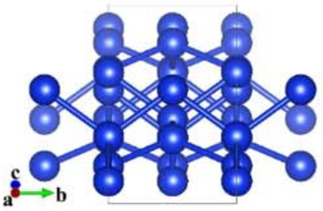

(31) 12-5-20-235525

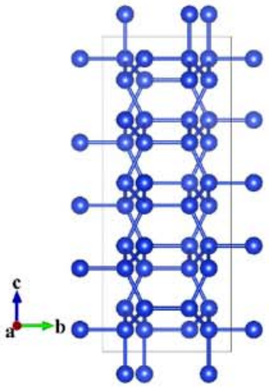

(32) 12-5-40-232334

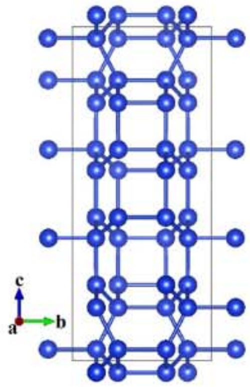

(33) 12-5-40-235831

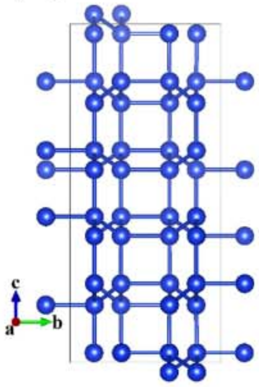

(34) 12-5-40-000356

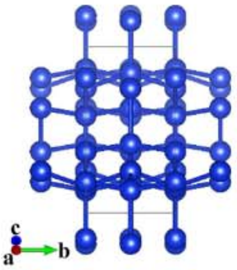

(35) 12-6-24-235351

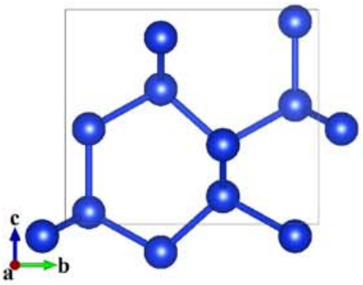

(36) 13-2-8-002729

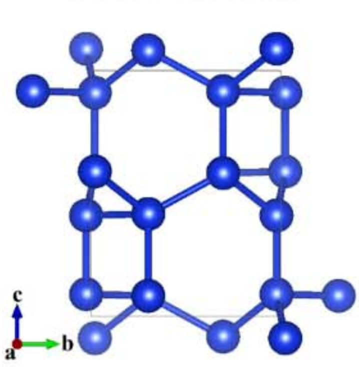

(37) 13-2-8-231921

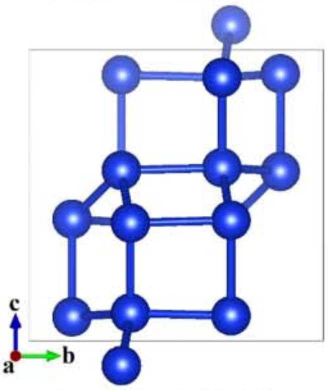

(38) 13-3-12-233948

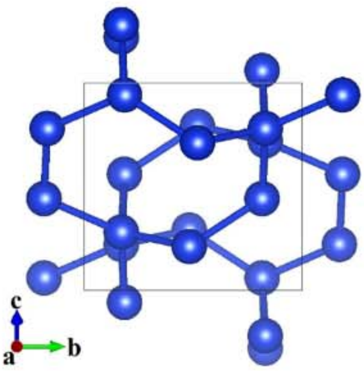

(39) 13-3-12-235231

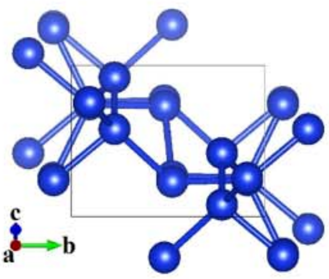

(40) 13-3-12-235342

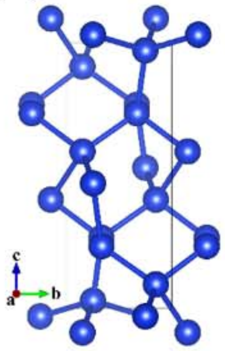

(41) 13-3-12-232304

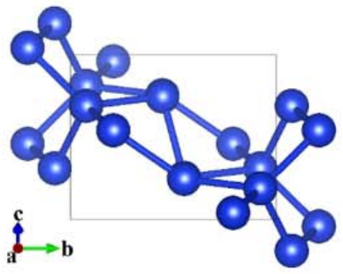

(42) 13-3-12-233214

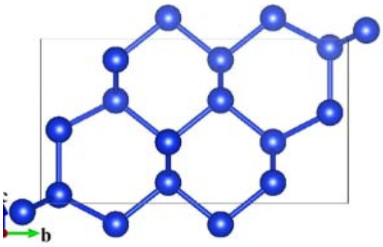

(43) 13-3-12-232202

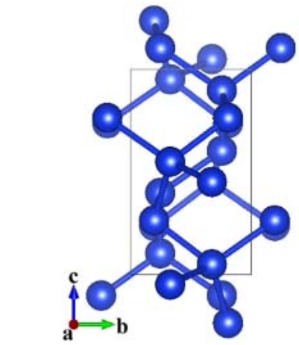

(44) 13-3-12-232508

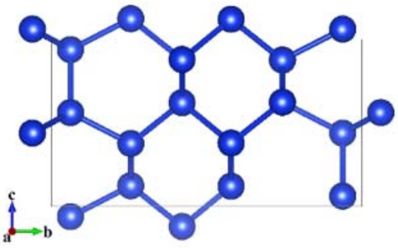

(45) 13-3-12-232717

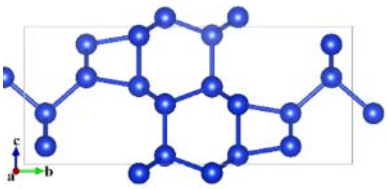

(46) 13-4-16-001718

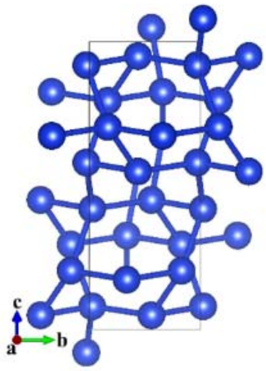

(47) 13-4-16-235907

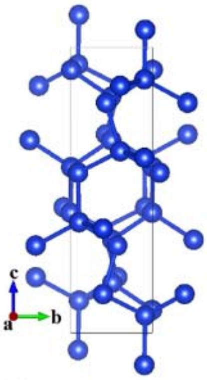

(48) 13-6-24-233744

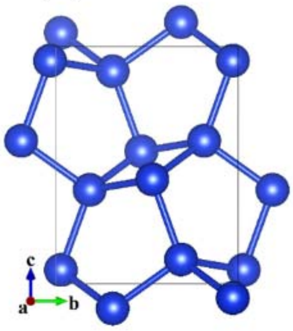

(49) 14-2-8-232118

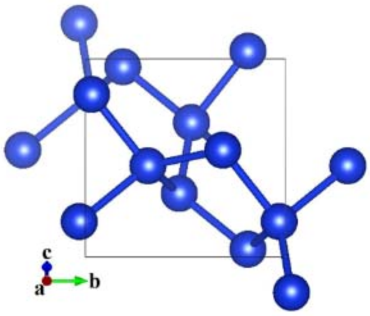

(50) 14-2-8-232552

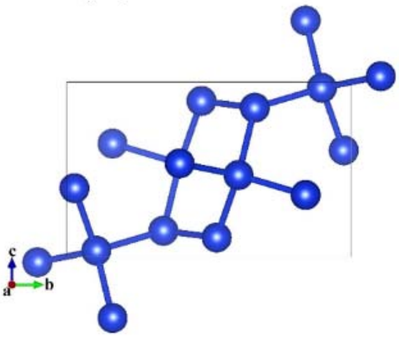

(51) 14-3-12-235423

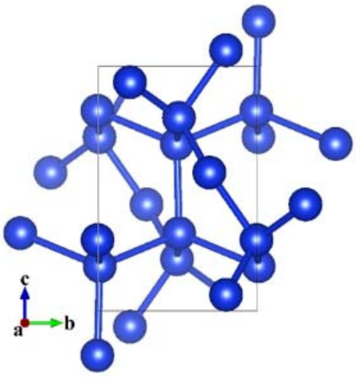

(52) 14-3-12--001732

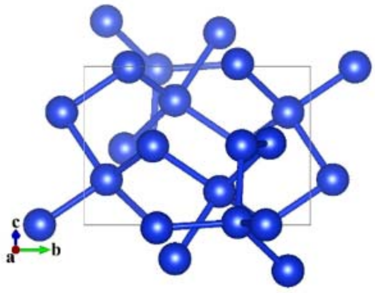

(53) 14-3-12-231751

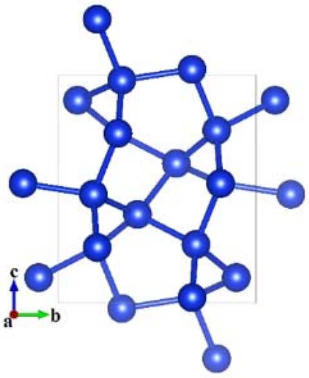

(54) 14-3-12-235501

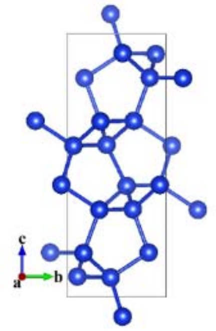
(55) 14-4-16-000223

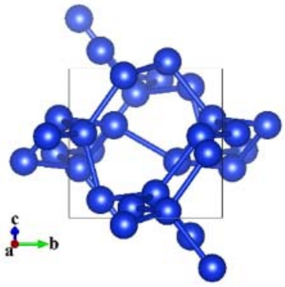
(56) 14-4-16-000321

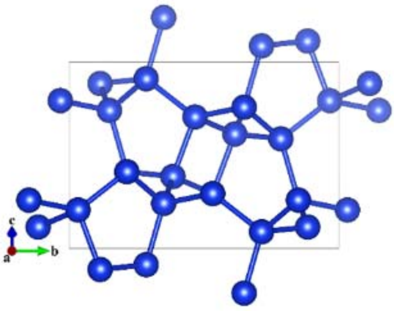
(57) 14-4-16-232843

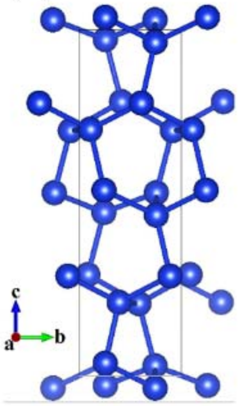
(58) 14-4-16-232103

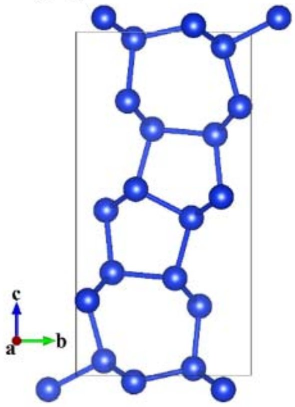
(59) 14-4-16-232446

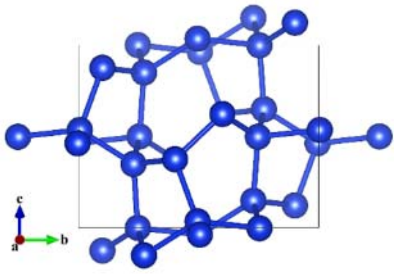
(60) 14-4-16-233726

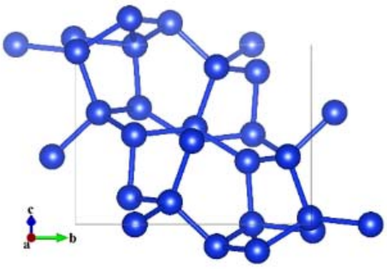
(61) 14-4-16-234845

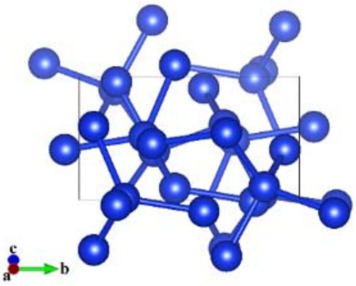
(62) 14-4-16-235340

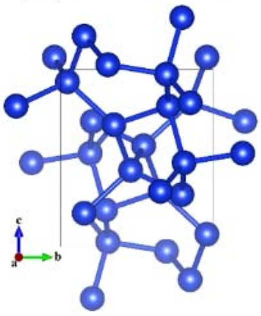
(63) 14-4-16-235403

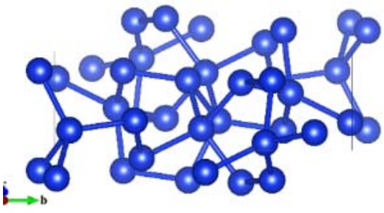
(64) 14-5-20-232541

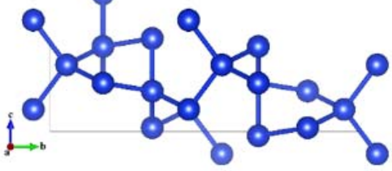
(65) 15-2-12-011815

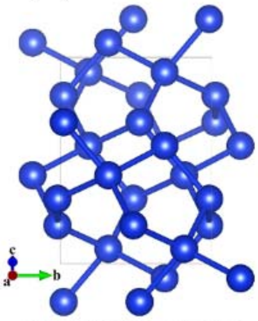
(66) 15-2-16-231909

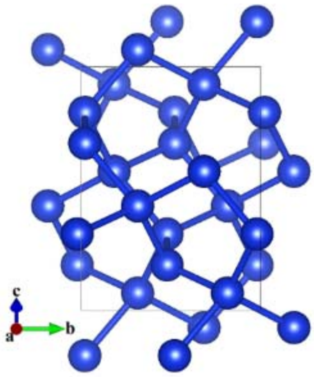
(67) 15-2-16-232558

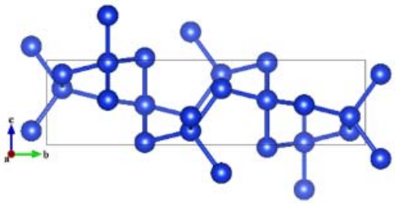
(68) 15-2-16-231933

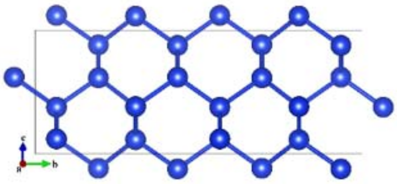
(69) 15-2-16-232050

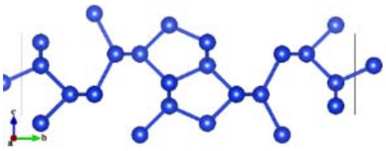
(70) 15-3-16-234156

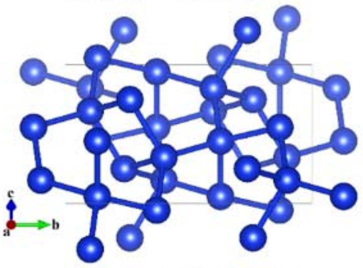
(71) 15-3-20-001715

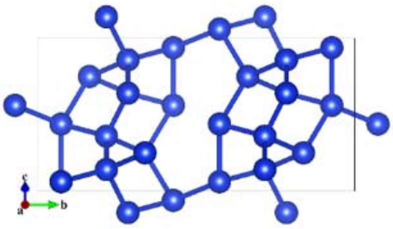
(72) 15-3-20-232008

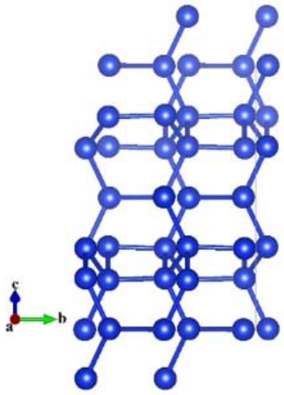
(73) 15-3-24-233430

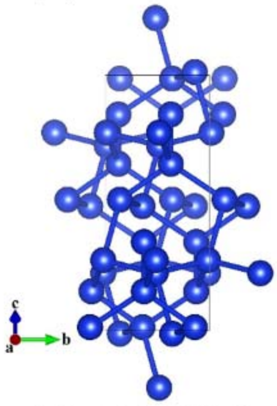
(74) 15-3-24-232148

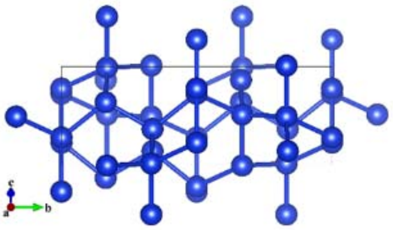
(75) 15-3-24-233315

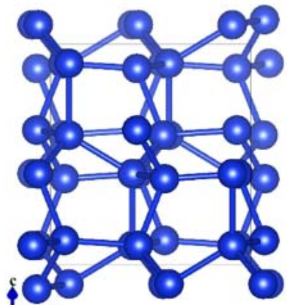
(76) 15-3-24-001219

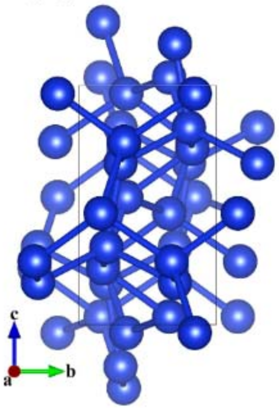
(77) 15-3-24-232448

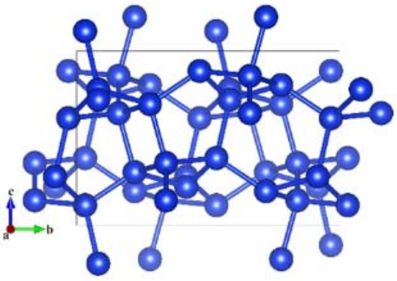
(78) 15-4-32-235759

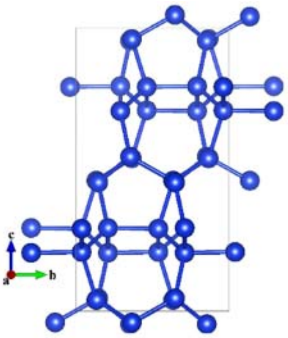

(79) 15-4-32-233050

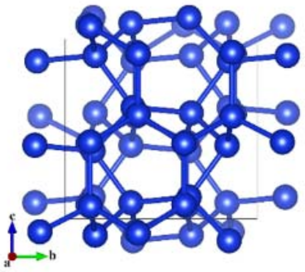

(80) 15-4-32-233356

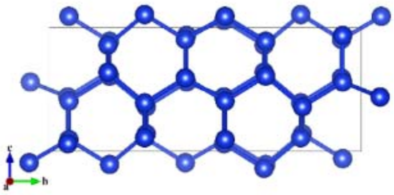

(81) 15-4-32-000249

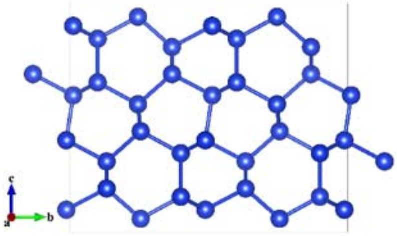

(82) 15-4-32-000324

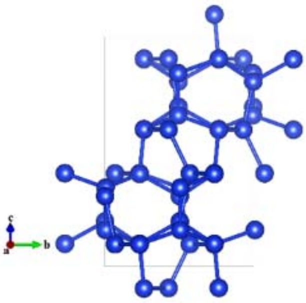

(83) 15-5-40-001314

Figure S2 The crystal structures of 83 new 3D sp^3 silicon allotropes in monoclinic phases.

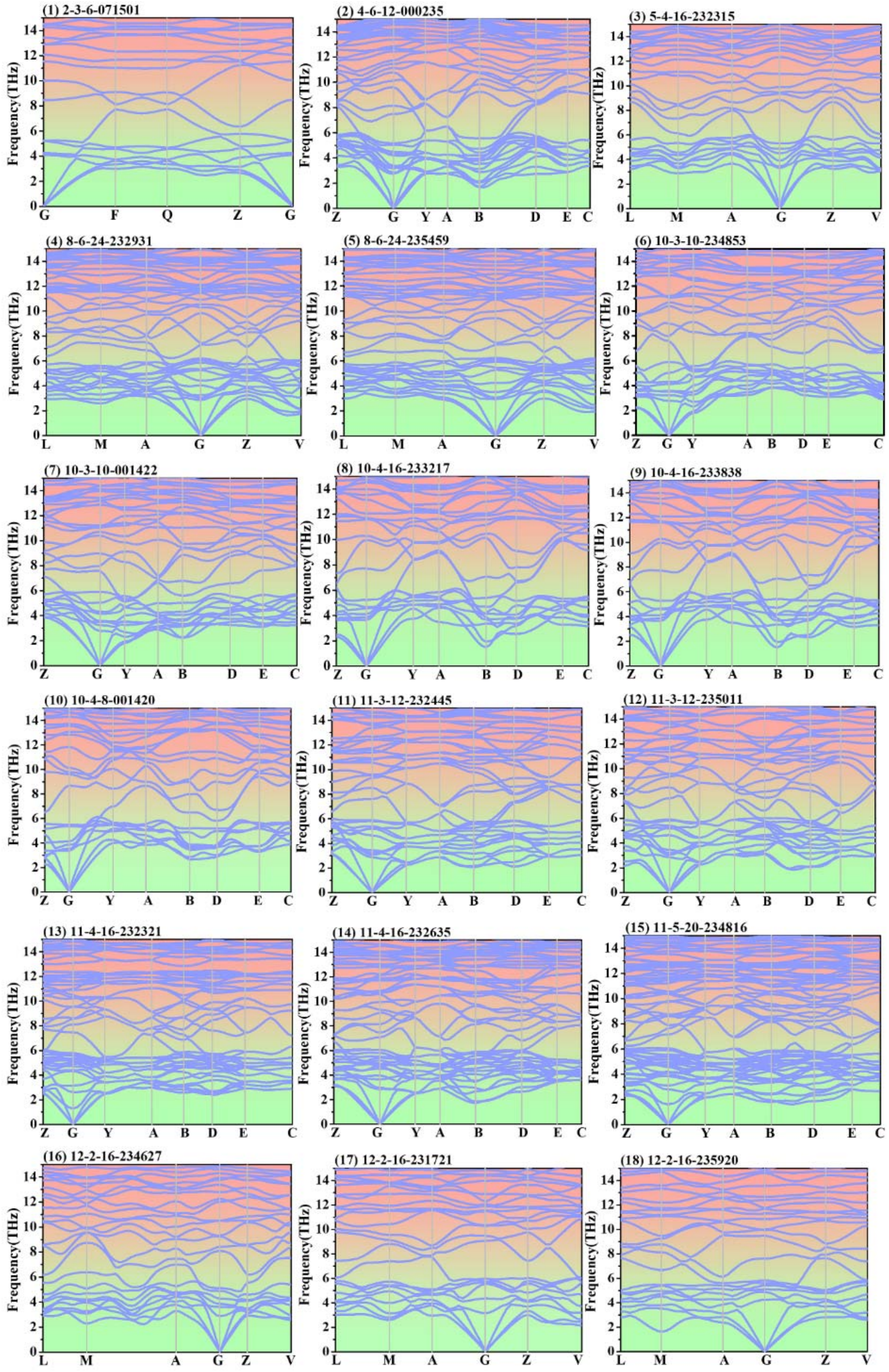
(1) 2-3-6-071501
Frequency(THz)
G F Q Z G
(2) 4-6-12-000235
Frequency(THz)
Z G Y A B D E C
(3) 5-4-16-232315
Frequency(THz)
L M A G Z V
(4) 8-6-24-232931
Frequency(THz)
L M A G Z V
(5) 8-6-24-235459
Frequency(THz)
L M A G Z V
(6) 10-3-10-234853
Frequency(THz)
Z G Y A B D E C
(7) 10-3-10-001422
Frequency(THz)
Z G Y A B D E C
(8) 10-4-16-233217
Frequency(THz)
Z G Y A B D E C
(9) 10-4-16-233838
Frequency(THz)
Z G Y A B D E C
(10) 10-4-8-001420
Frequency(THz)
Z G Y A B D E C
(11) 11-3-12-232445
Frequency(THz)
Z G Y A B D E C
(12) 11-3-12-235011
Frequency(THz)
Z G Y A B D E C
(13) 11-4-16-232321
Frequency(THz)
Z G Y A B D E C
(14) 11-4-16-232635
Frequency(THz)
Z G Y A B D E C
(15) 11-5-20-234816
Frequency(THz)
Z G Y A B D E C
(16) 12-2-16-234627
Frequency(THz)
L M A G Z V
(17) 12-2-16-231721
Frequency(THz)
L M A G Z V
(18) 12-2-16-235920
Frequency(THz)
L M A G Z V

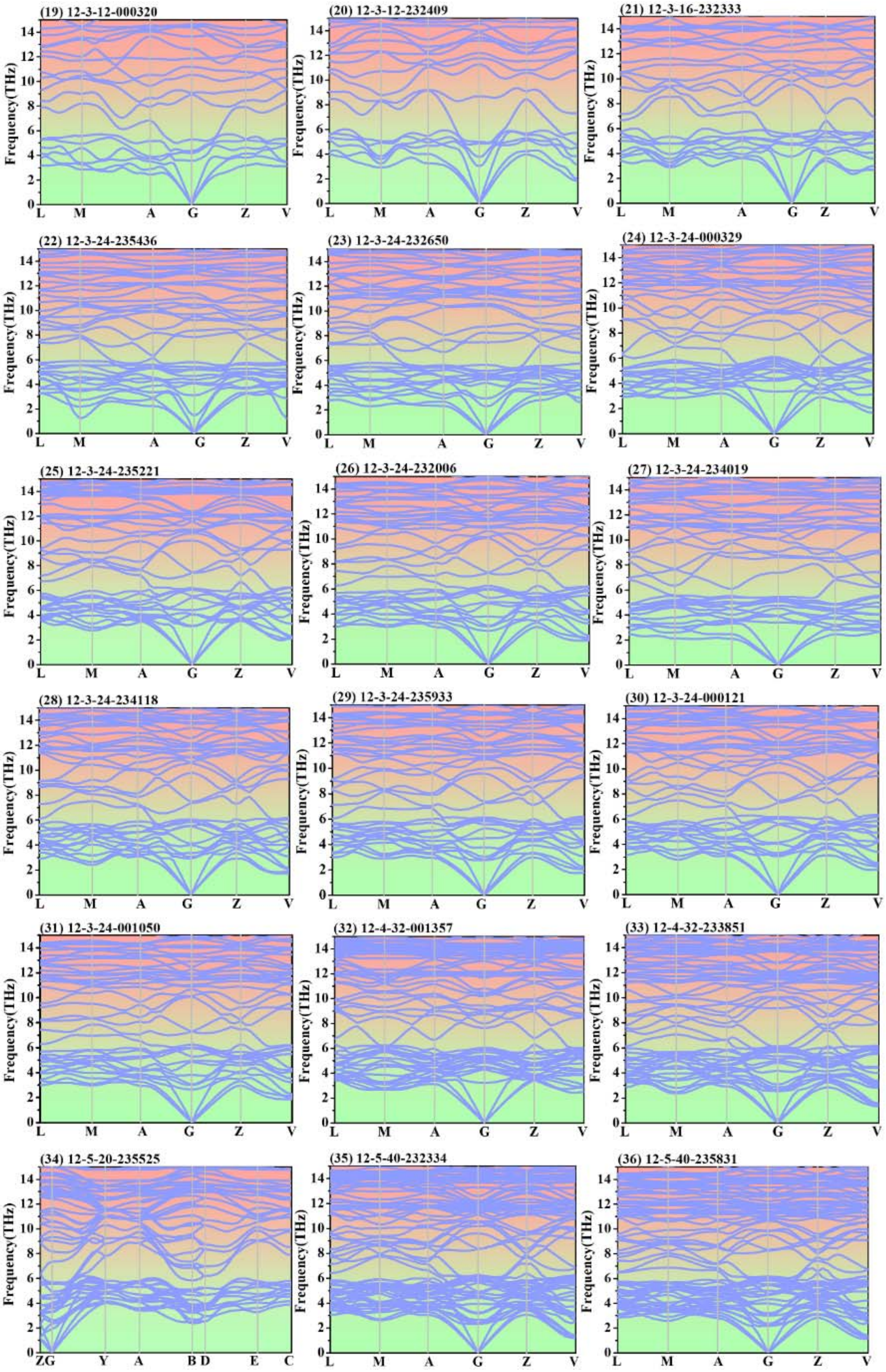
(19) 12-3-12-000320
(20) 12-3-12-232409
(21) 12-3-16-232333
(22) 12-3-24-235436
(23) 12-3-24-232650
(24) 12-3-24-000329
(25) 12-3-24-235221
(26) 12-3-24-232006
(27) 12-3-24-234019
(28) 12-3-24-234118
(29) 12-3-24-235933
(30) 12-3-24-000121
(31) 12-3-24-001050
(32) 12-4-32-001357
(33) 12-4-32-233851
(34) 12-5-20-235525
(35) 12-5-40-232334
(36) 12-5-40-235831
Frequency(THz)
L M A G Z V
ZG Y A B D E C

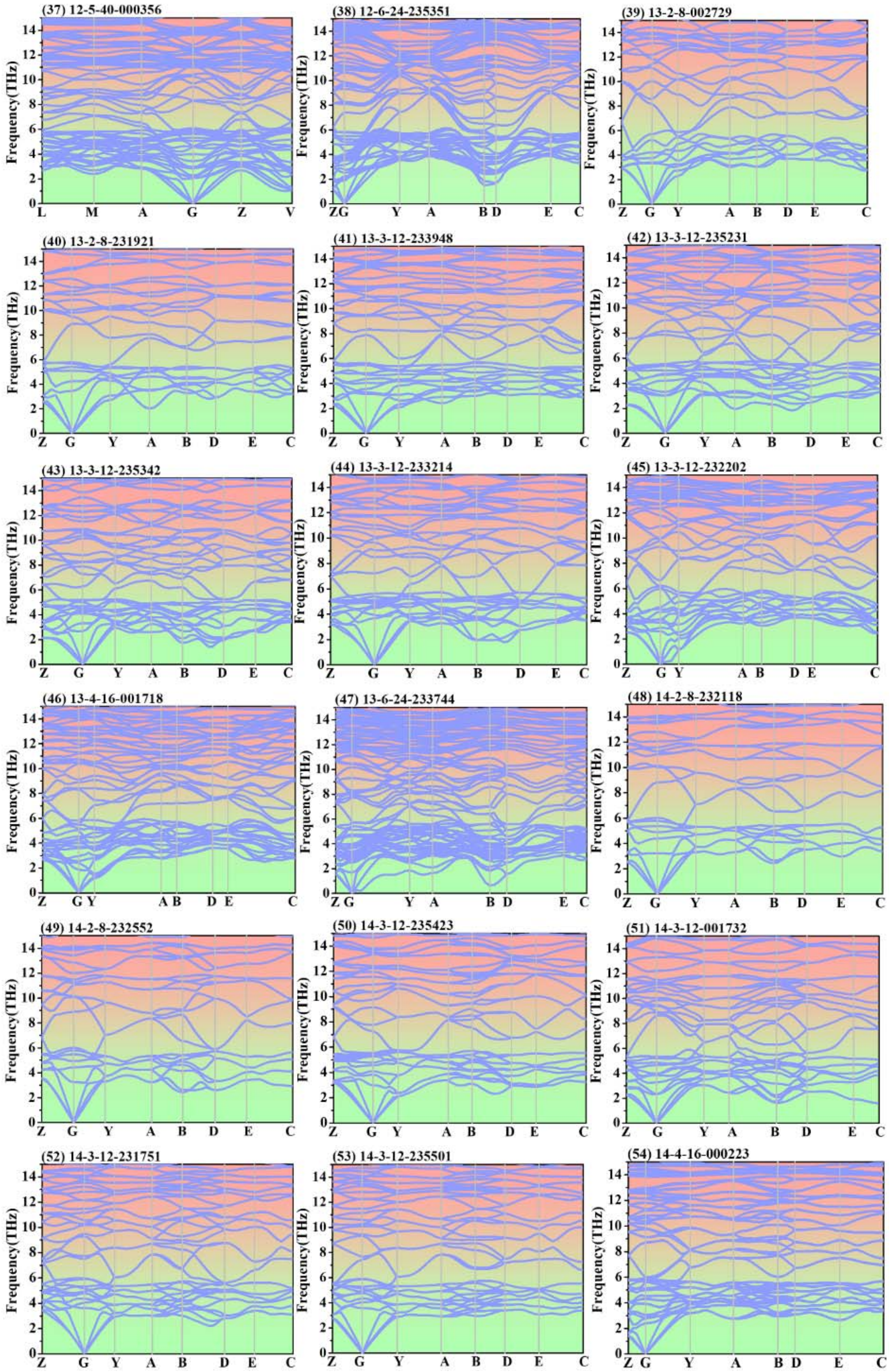
(37) 12-5-40-000356
(38) 12-6-24-235351
(39) 13-2-8-002729
(40) 13-2-8-231921
(41) 13-3-12-233948
(42) 13-3-12-235231
(43) 13-3-12-235342
(44) 13-3-12-233214
(45) 13-3-12-232202
(46) 13-4-16-001718
(47) 13-6-24-233744
(48) 14-2-8-232118
(49) 14-2-8-232552
(50) 14-3-12-235423
(51) 14-3-12-001732
(52) 14-3-12-231751
(53) 14-3-12-235501
(54) 14-4-16-000223
Frequency(THz)
L M A G Z V
ZG Y A B D E C
Z G Y A B D E C

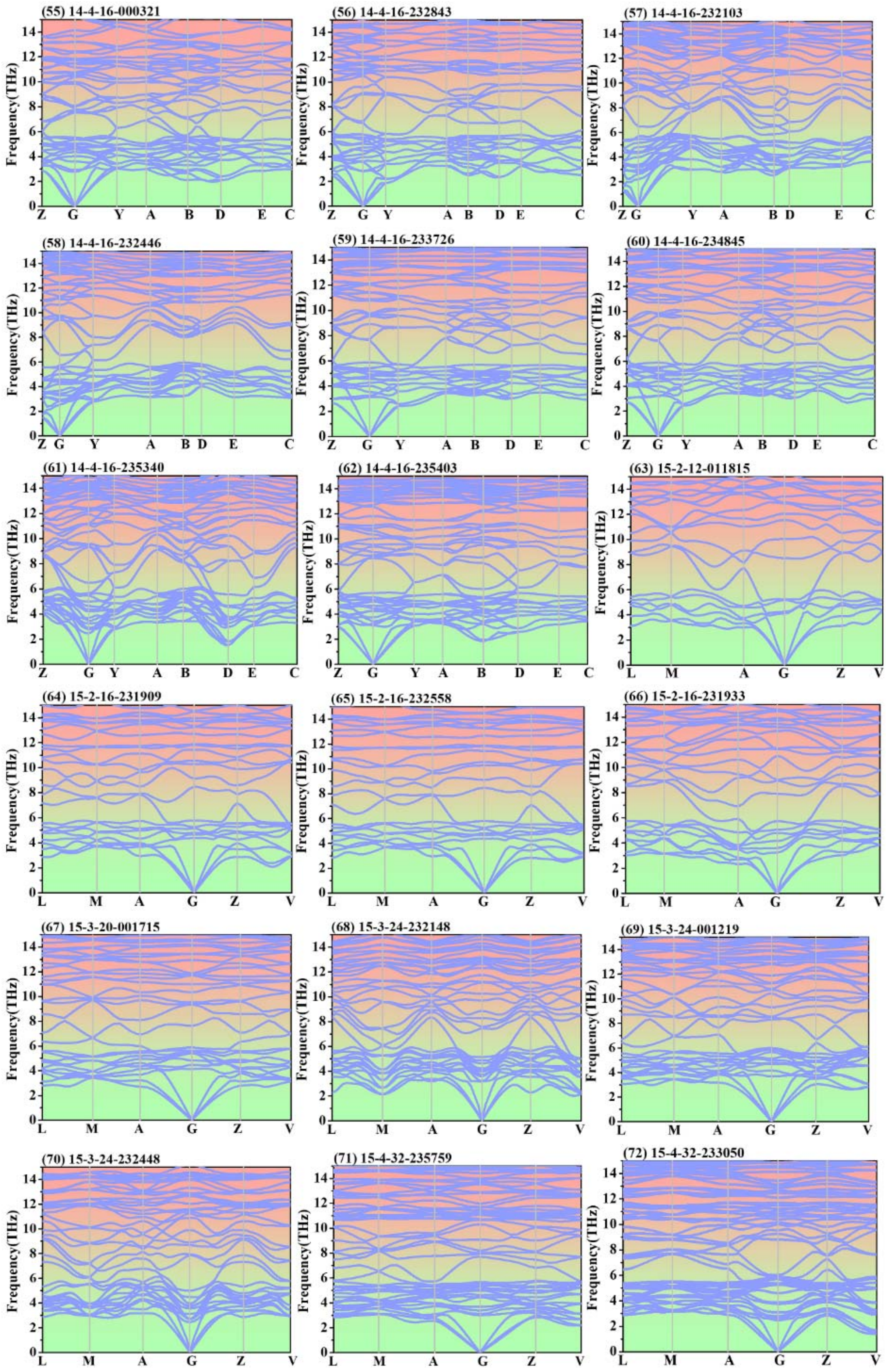
(55) 14-4-16-000321
Frequency(THz)
Z G Y A B D E C
(56) 14-4-16-232843
Z G Y A B D E C
(57) 14-4-16-232103
Z G Y A B D E C
(58) 14-4-16-232446
Z G Y A B D E C
(59) 14-4-16-233726
Z G Y A B D E C
(60) 14-4-16-234845
Z G Y A B D E C
(61) 14-4-16-235340
Z G Y A B D E C
(62) 14-4-16-235403
Z G Y A B D E C
(63) 15-2-12-011815
L M A G Z V
(64) 15-2-16-231909
L M A G Z V
(65) 15-2-16-232558
L M A G Z V
(66) 15-2-16-231933
L M A G Z V
(67) 15-3-20-001715
L M A G Z V
(68) 15-3-24-232148
L M A G Z V
(69) 15-3-24-001219
L M A G Z V
(70) 15-3-24-232448
L M A G Z V
(71) 15-4-32-235759
L M A G Z V
(72) 15-4-32-233050
L M A G Z V

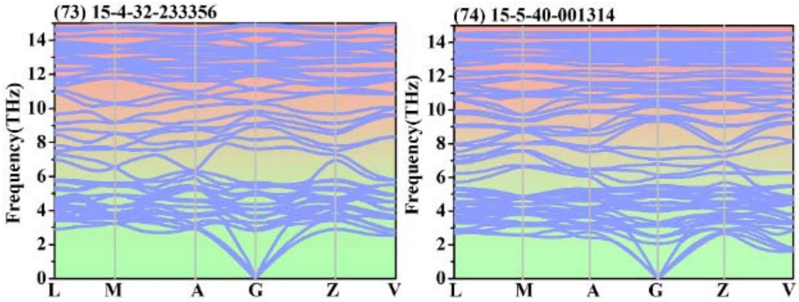

Figure S3 Phonon spectra of new 3D sp^3 silicon allotropes in monoclinic phases.

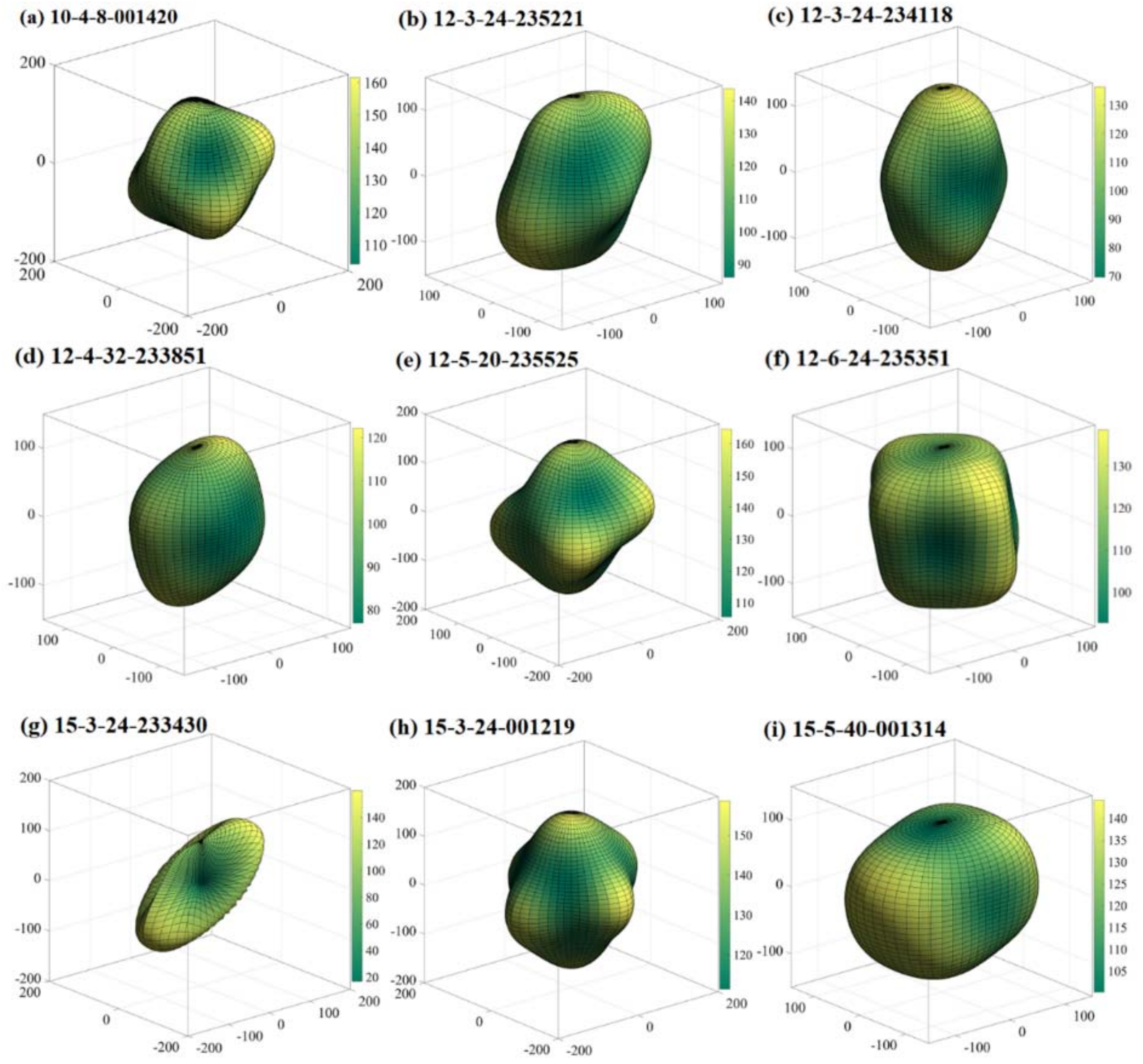

Figure S4 Three-dimensional plots of Young's moduli of novel silicon allotropes with quasi-direct band gap.

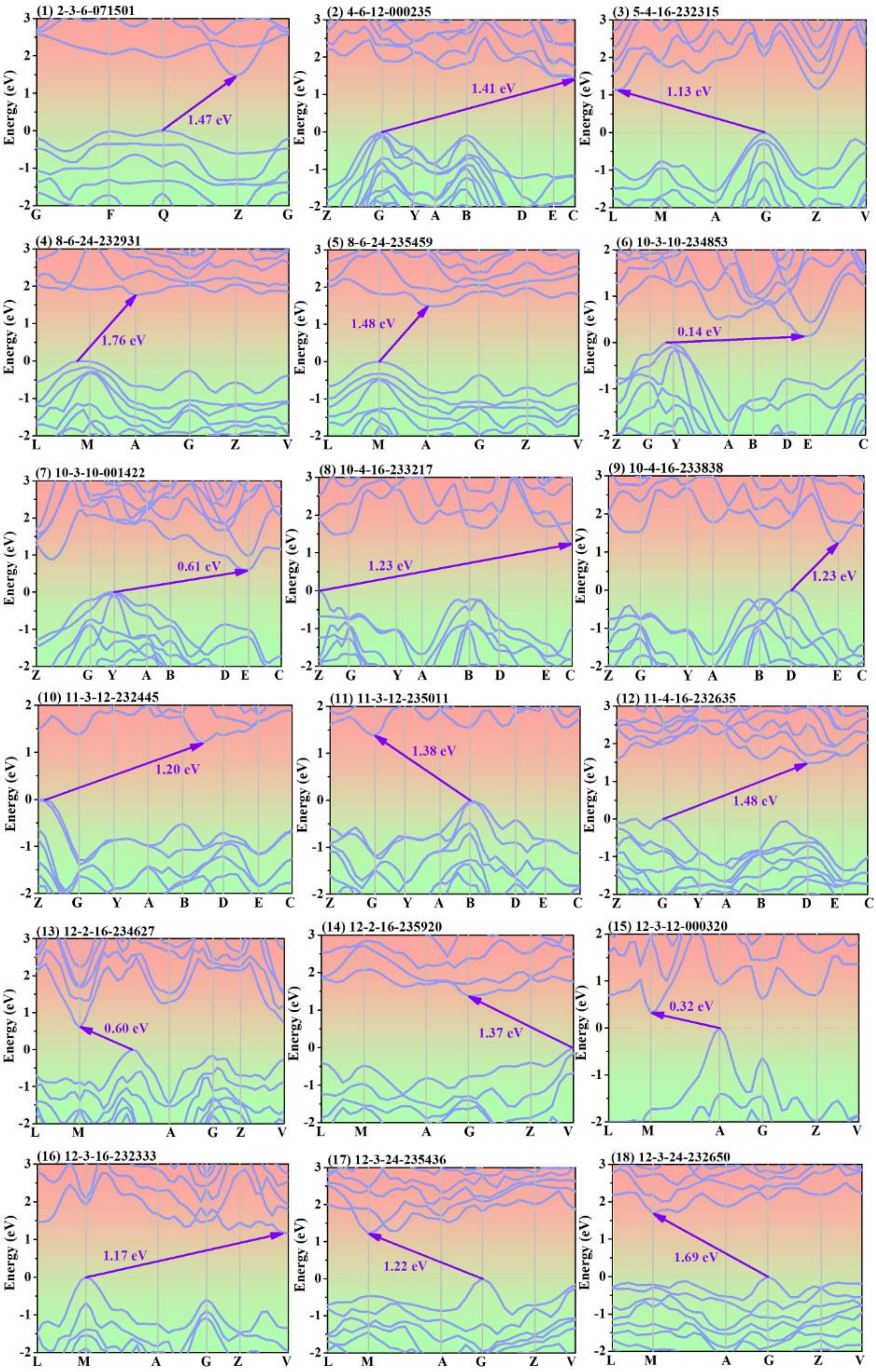
(1) 2-3-6-071501
1.47 eV
G F Q Z G
(2) 4-6-12-000235
1.41 eV
Z G Y A B D E C
(3) 5-4-16-232315
1.13 eV
L M A G Z V
(4) 8-6-24-232931
1.76 eV
L M A G Z V
(5) 8-6-24-235459
1.48 eV
L M A G Z V
(6) 10-3-10-234853
0.14 eV
Z G Y A B D E C
(7) 10-3-10-001422
0.61 eV
Z G Y A B D E C
(8) 10-4-16-233217
1.23 eV
Z G Y A B D E C
(9) 10-4-16-233838
1.23 eV
Z G Y A B D E C
(10) 11-3-12-232445
1.20 eV
Z G Y A B D E C
(11) 11-3-12-235011
1.38 eV
Z G Y A B D E C
(12) 11-4-16-232635
1.48 eV
Z G Y A B D E C
(13) 12-2-16-234627
0.60 eV
L M A G Z V
(14) 12-2-16-235920
1.37 eV
L M A G Z V
(15) 12-3-12-000320
0.32 eV
L M A G Z V
(16) 12-3-16-232333
1.17 eV
L M A G Z V
(17) 12-3-24-235436
1.22 eV
L M A G Z V
(18) 12-3-24-232650
1.69 eV
L M A G Z V
Energy (eV)

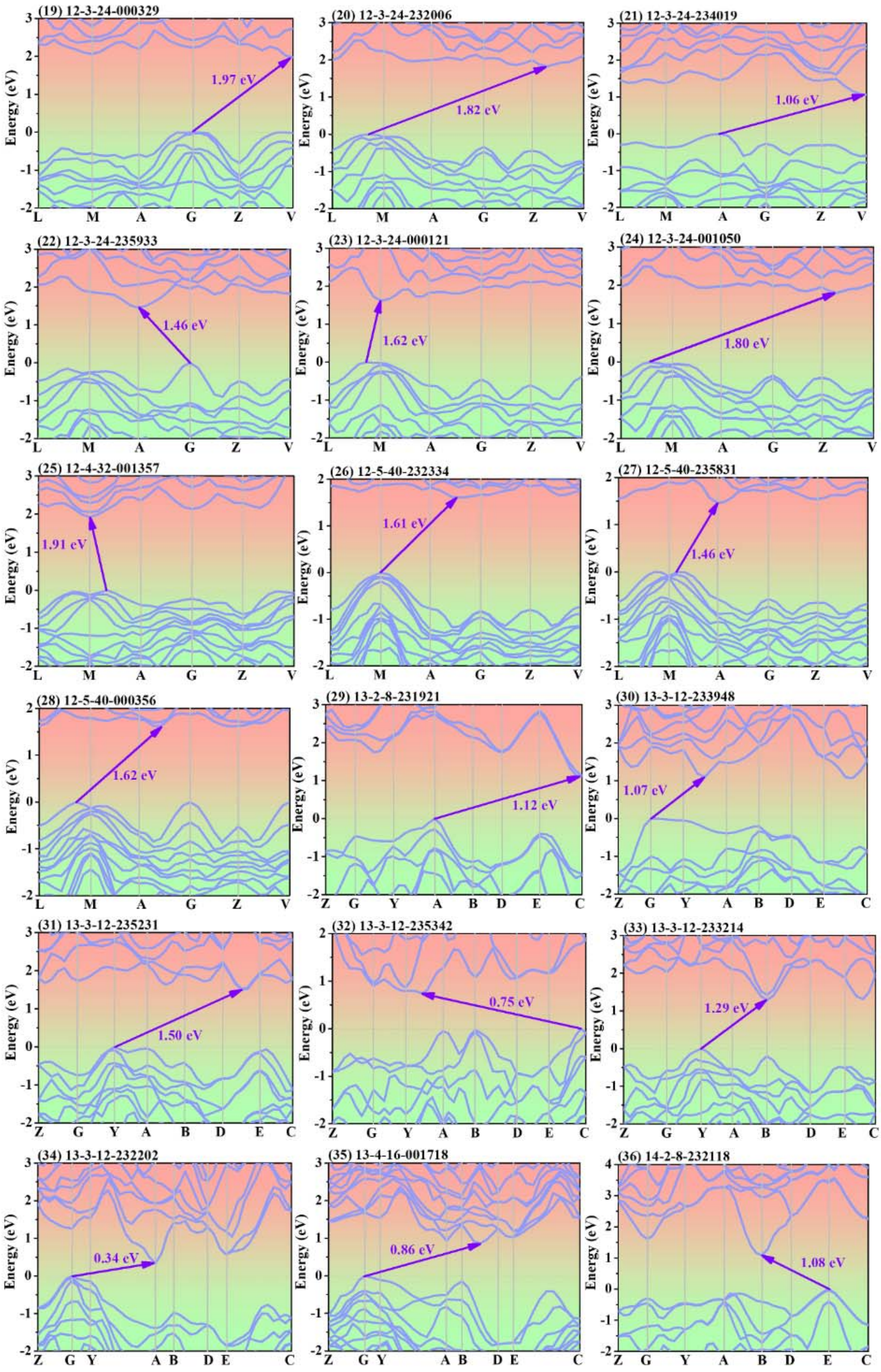
(19) 12-3-24-000329
1.97 eV
(20) 12-3-24-232006
1.82 eV
(21) 12-3-24-234019
1.06 eV
(22) 12-3-24-235933
1.46 eV
(23) 12-3-24-000121
1.62 eV
(24) 12-3-24-001050
1.80 eV
(25) 12-4-32-001357
1.91 eV
(26) 12-5-40-232334
1.61 eV
(27) 12-5-40-235831
1.46 eV
(28) 12-5-40-000356
1.62 eV
(29) 13-2-8-231921
1.12 eV
(30) 13-3-12-233948
1.07 eV
(31) 13-3-12-235231
1.50 eV
(32) 13-3-12-235342
0.75 eV
(33) 13-3-12-233214
1.29 eV
(34) 13-3-12-232202
0.34 eV
(35) 13-4-16-001718
0.86 eV
(36) 14-2-8-232118
1.08 eV
Energy (eV)

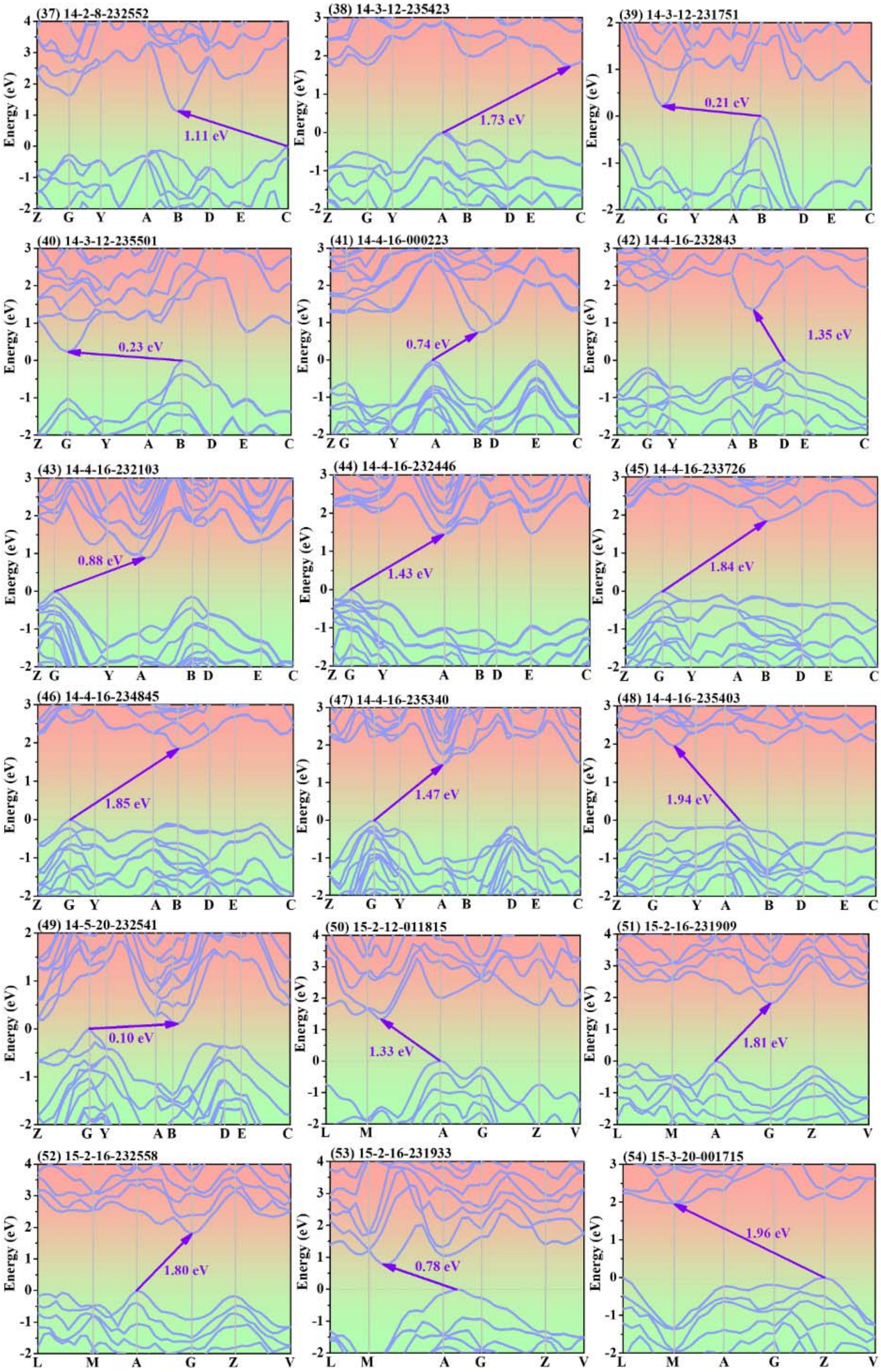
(37) 14-2-8-232552
1.11 eV
(38) 14-3-12-235423
1.73 eV
(39) 14-3-12-231751
0.21 eV
(40) 14-3-12-235501
0.23 eV
(41) 14-4-16-000223
0.74 eV
(42) 14-4-16-232843
1.35 eV
(43) 14-4-16-232103
0.88 eV
(44) 14-4-16-232446
1.43 eV
(45) 14-4-16-233726
1.84 eV
(46) 14-4-16-234845
1.85 eV
(47) 14-4-16-235340
1.47 eV
(48) 14-4-16-235403
1.94 eV
(49) 14-5-20-232541
0.10 eV
(50) 15-2-12-011815
1.33 eV
(51) 15-2-16-231909
1.81 eV
(52) 15-2-16-232558
1.80 eV
(53) 15-2-16-231933
0.78 eV
(54) 15-3-20-001715
1.96 eV
Energy (eV)

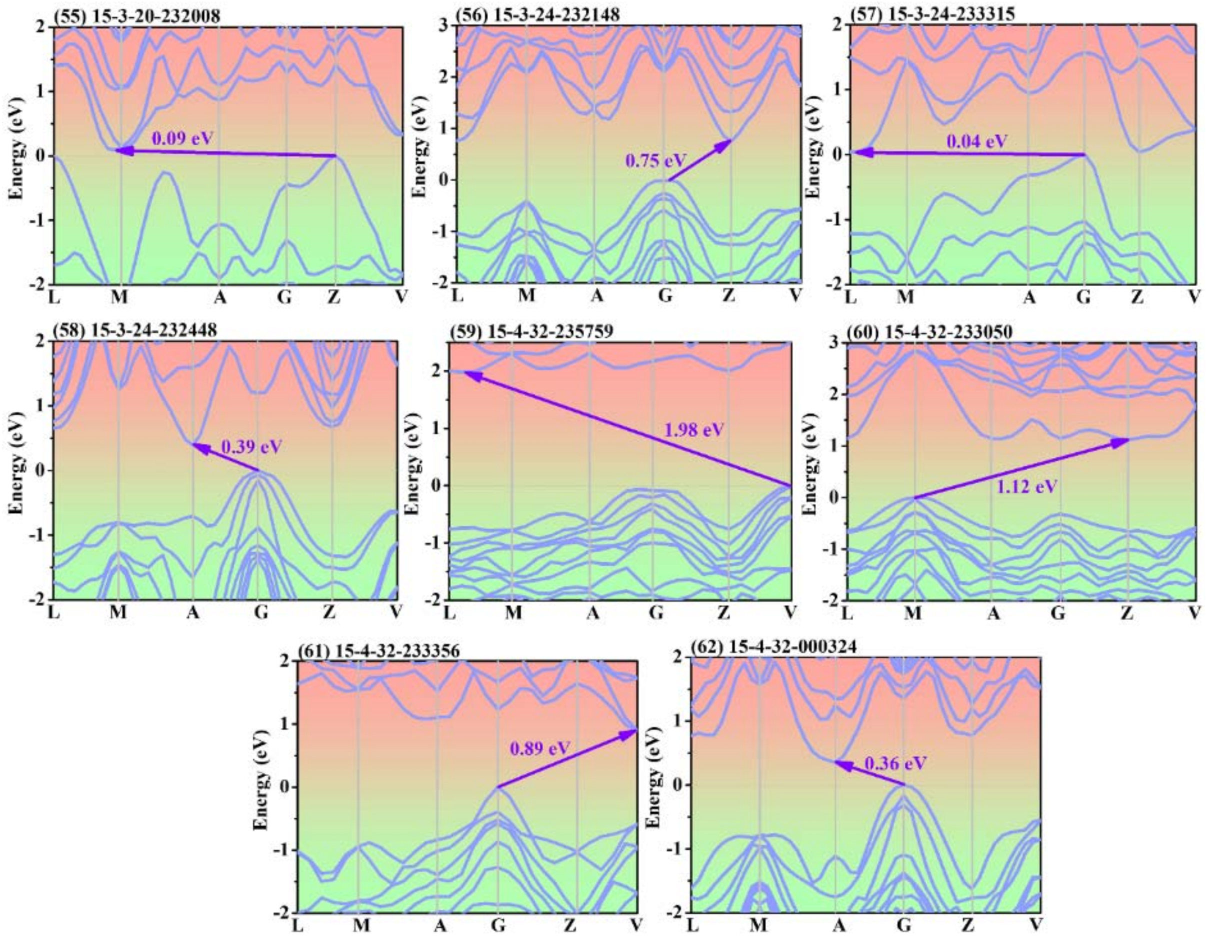

Figure S5 The electronic band structures of 62 novel silicon allotropes with semiconductor properties in monoclinic symmetry.

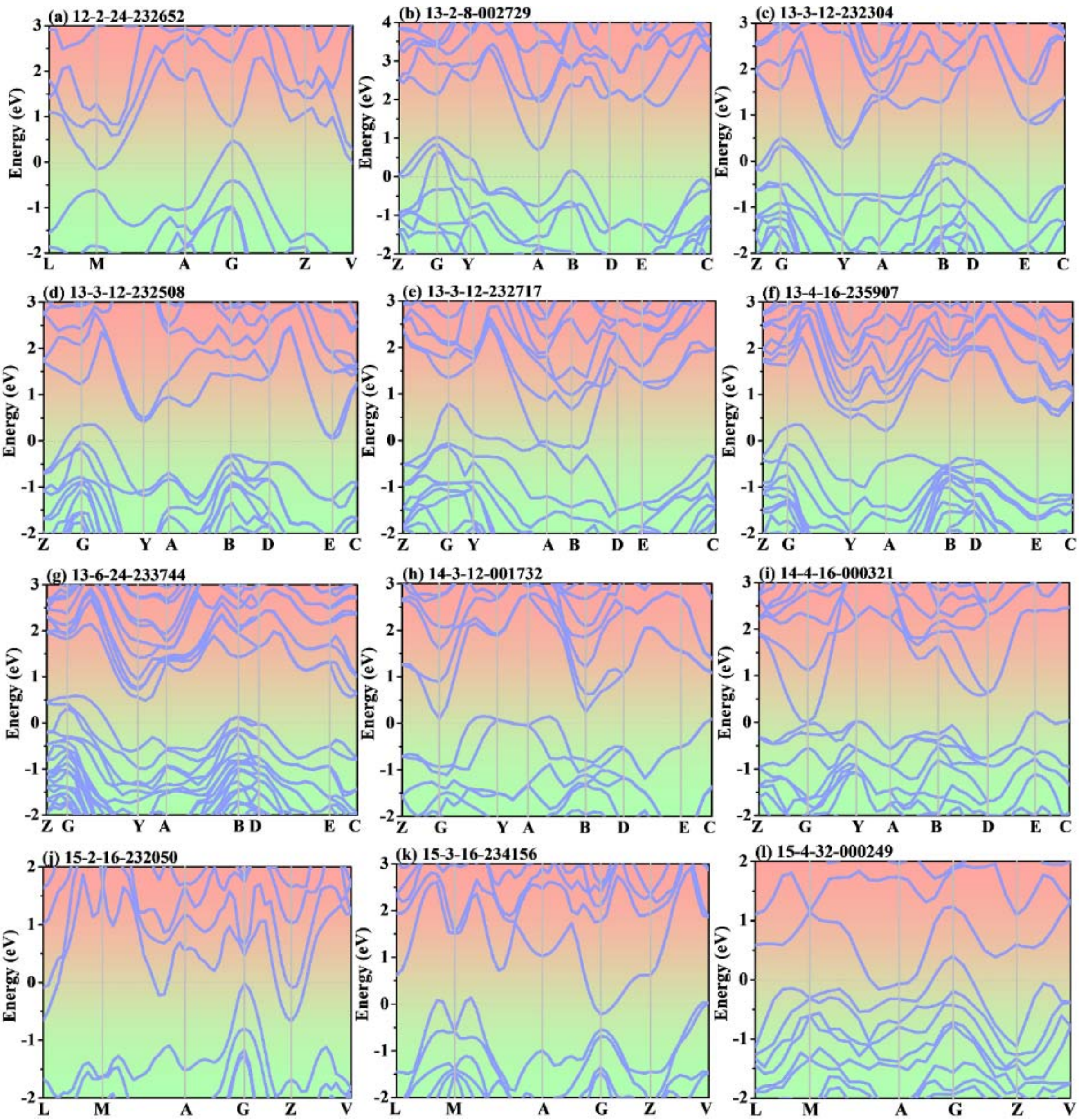

Figure S6 The electronic band structures of 12 silicon allotropes with metallic properties in monoclinic symmetry.

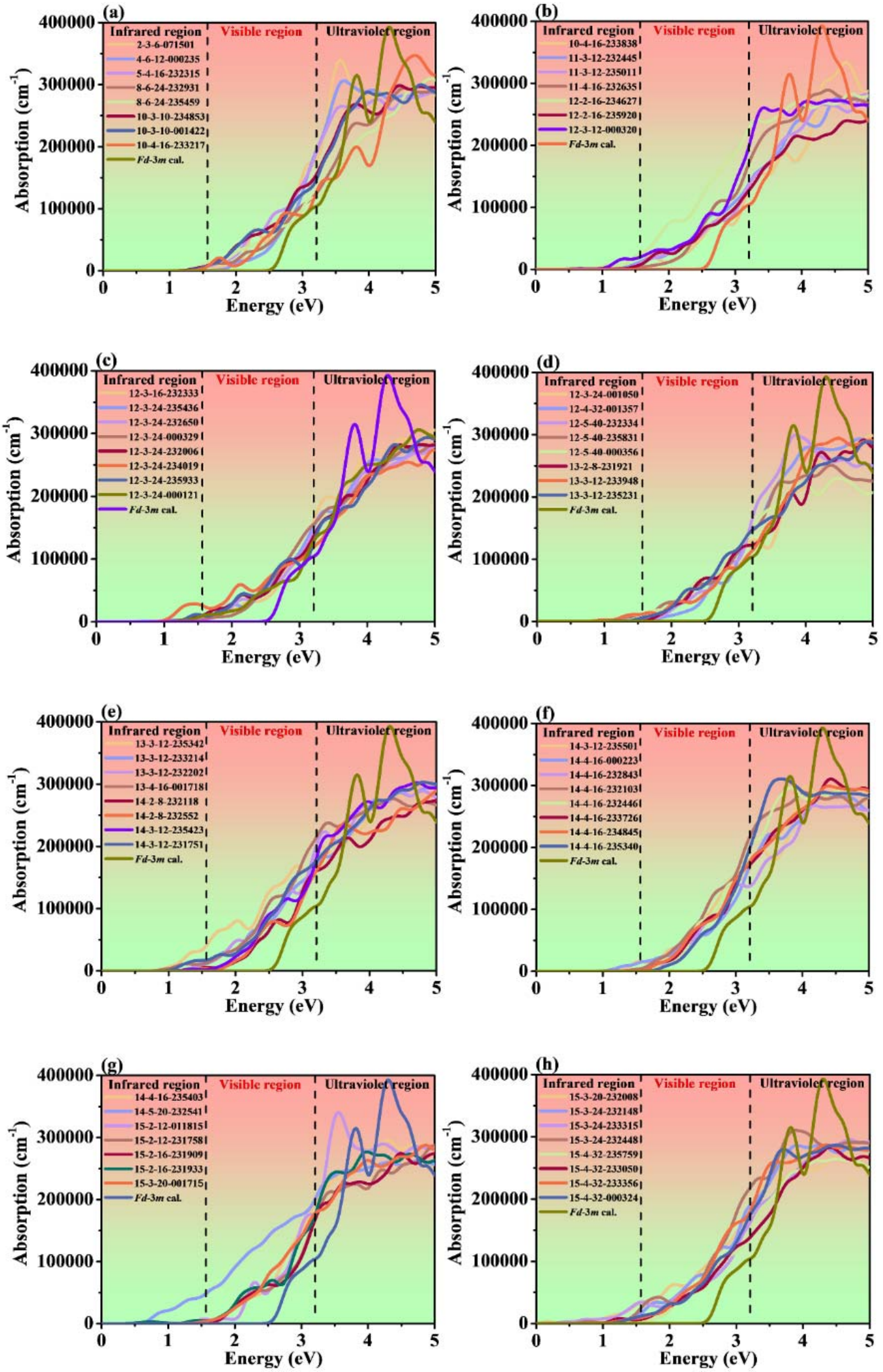

Figure S7 The absorption spectra of 62 novel silicon allotropes with semiconductor properties in monoclinic symmetry within the GGA-PBE level.
